# The formation of unsaturated IrO_x_ in SrIrO_3_ by cobalt-doping for acidic oxygen evolution reaction

**DOI:** 10.1038/s41467-024-46801-y

**Published:** 2024-04-04

**Authors:** Jia-Wei Zhao, Kaihang Yue, Hong Zhang, Shu-Yin Wei, Jiawei Zhu, Dongdong Wang, Junze Chen, Vyacheslav Yu. Fominski, Gao-Ren Li

**Affiliations:** 1 College of Materials Science and Engineering, Sichuan University, Chengdu, 610065 China; 2 School of Chemistry, Sun Yat-sen University, Guangzhou, 510275 China; 3 Department of Mechanical Engineering, City University of Hong Kong, 83 Tat Chee Avenue, Kowloon, Hong Kong SAR, 999077 China; 4 CAS Key Laboratory of Materials for Energy Conversion, Shanghai Institute of Ceramics, Chinese Academy of Sciences (SICCAS), 585 Heshuo Road, Shanghai, 200050 China; 5 Electron Microscopy Centre, School of Physical Science and Technology, Lanzhou University, Lanzhou, 730099 China; 6 National Research Nuclear University MEPhI (Moscow Engineering Physics Institute), Kashirskoe sh. 31, Moscow, 115409 Russia

**Keywords:** Electrocatalysis, Electrocatalysis, Electronic properties and materials

## Abstract

Electrocatalytic water splitting is a promising route for sustainable hydrogen production. However, the high overpotential of the anodic oxygen evolution reaction poses significant challenge. SrIrO_3_-based perovskite-type catalysts have shown great potential for acidic oxygen evolution reaction, but the origins of their high activity are still unclear. Herein, we develop a Co-doped SrIrO_3_ system to enhance oxygen evolution reaction activity and elucidate the origin of catalytic activity. In situ experiments reveal Co activates surface lattice oxygen, rapidly exposing IrO_x_ active sites, while bulk Co doping optimizes the adsorbate binding energy of IrO_x_. The Co-doped SrIrO_3_ demonstrates high oxygen evolution reaction electrocatalytic activity, markedly surpassing the commercial IrO_2_ catalysts in both conventional electrolyzer and proton exchange membrane water electrolyzer.

## Abstract (web summary)

SrIrO_3_-based catalyst is a promising candidate for acidic oxygen evolution reaction (OER), but the active sites and factors responsible for its effectiveness remain ambiguous. Here, the authors reveal that dynamic dissolution of Co in Co-SrIrO_3_ leads to the formation of unsaturated IrOx, enabling high-efficiency OER.

## Introduction

Water splitting for hydrogen production offers the advantage of producing clean and sustainable fuel without carbon emissions^1,2^. To date, proton exchange membrane (PEM) water electrolysis is one of the most established ways in the field of green hydrogen production^3–5^. However, the high overpotential typically associated with the anodic oxygen evolution reaction (OER) poses a significant challenge to enhancing hydrogen production efficiency from water electrolysis^6^. Furthermore, the advancement of OER catalysts designed for acidic medium poses a greater challenge compared to those intended for alkaline medium. Since highly OER-active electrocatalysts are mainly comprised of metal oxides or hydroxides, most of which exhibit poor stability under acidic conditions^7–10^. Therefore, the development of efficient OER catalysts functioning in acidic medium is critical.

Current, effective acidic OER catalysts include Ru, Ir, and Mn-based metal oxides^11–17^. In particular, SrIrO_3_ with an ABO_3_ perovskite structure (where A typically represents an alkaline earth metal and B represents a transition metal) shows high acidic OER catalytic performance^18–30^. In recent years, extensive researchers have been conducted to understand the OER catalytic mechanism of SrIrO_3_. Jaramillo et al. prepared the (001) plane SrIrO_3_ film via a laser epitaxy strategy, and observed an incremental improvement in its OER catalytic performance throughout the catalytic process^22^. Through combining theoretical calculations and experiments, they discovered that the high catalytic activity of SrIrO_3_ could be attributed to the exposed IrO_x_ sites following Sr dissolution. In further comprehensive studies, researchers confirmed the structural modifications of SrIrO_3_ under acidic OER process using secondary ion mass spectrometry (SIMS), in situ atomic force microscopy (AFM), and X-ray absorption spectroscopy (XAS), respectively^23–25^. They proposed a correlation between the Sr dissolution process and the formed IrO_x_ surface activities. Moreover, an investigation involving SrIr_0.1_Co_0.9_O_3_ further indicates that the OER activity also originates from the amorphous IrO_x_ structure formed by the dissolution of Co^26^. These studies appear to identify the active component of SrIrO_3_-based perovskites as the amorphous IrO_x_ structure. Nevertheless, most studies overlook the impact of B-site dissolution on surface oxygen stability and Ir-O coordination structure, the precise reasons contributing to the high OER catalytic performance of Co-doped SrIrO_3_ catalysts remain unclear. This issue primarily stems from the challenge faced by researchers in elucidating two key aspects of the OER catalytic process: (1) The key role of Co dissolution in catalytic processes and (2) the influence of surface and bulk Co on IrO_x_ sites.

**Fig. 1:**
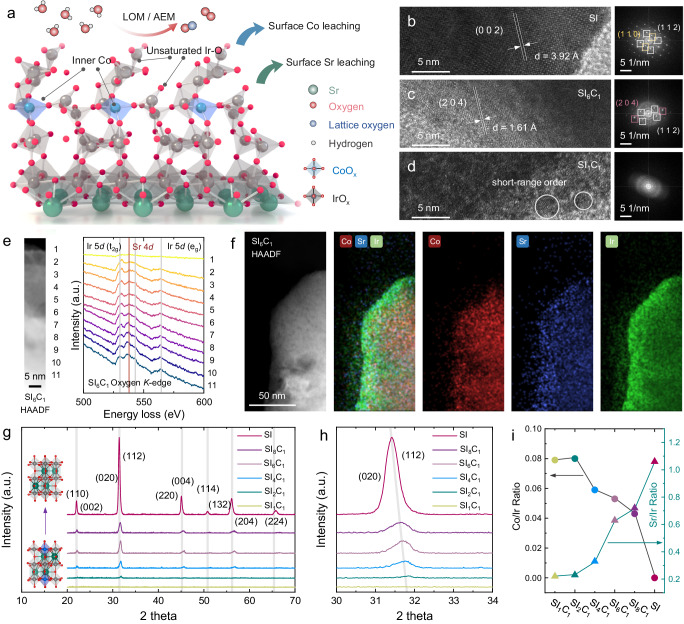
Characterizations of samples. **a** OER catalytic mechanism diagram of Co-doped SrIrO_3_ catalyst; **b**–**d** HRTEM images and corresponding FFT diagrams of SI, SI_6_C_1_ and SI_1_C_1_; **e** O *K*-edge EELS spectra of SI_6_C_1_; **f** HAADF image of SI_6_C_1_ and corresponding EDS mapping images; **g**, **h** XRD spectra of SI_1_C_1_, SI_2_C_1_, SI_4_C_1_, SI_6_C_1_, SI_8_C_1_, and SI samples. **i** ICP–MS diagram of Co/Ir and Sr/Ir ratios of SI_1_C_1_, SI_2_C_1_, SI_4_C_1_, SI_6_C_1_, SI_8_C_1_, and SI samples.

To address the two key challenges mentioned above, we designed a B-site Co-doped SrIrO_3_ system to discern the dissolution mechanism at catalyst sites and the origins of IrO_x_ catalytic activity, as shown in Fig. 1a. In situ inductively coupled plasma mass spectrometry (ICP-MS) experiments revealed simultaneous ion dissolution of Co and Sr, caused by acid corrosion prior to OER process. At the OER potential (1.60 V vs. RHE), the dissolution phenomenon was found to be negligible. Along with theoretical calculations, a series of in situ experiments including in situ Raman mapping, in situ XAS, and differential electrochemical mass spectrometry (DEMS) were conducted. These results highlighted the role of Co in two critical ways: (1) Surface Co reduces the stability of the Co-O-Ir bridge oxygen in SrIrO_3_, leading to the rapid exposure of the low-coordination IrO_x_ structure; (2) Bulk lattice Co optimizes the OOH binding energy of IrO_x_, consequently reducing the overpotential. Thus, the synthesized Co-doped SrIrO_3_ demonstrated high OER activity, markedly surpassing commercial IrO_2_ catalysts in PEM water electrolyzer. The insights obtained from this research would significantly enhance the understanding of high OER catalytic performance of SrIrO_3_-based perovskite catalysts, providing key insights for designing and preparing high-performance acidic OER catalysts.

## Results

### Fabrication and characterizations of Co-doped SrIrO_3_ samples

SrIrO_3_ doped with different amounts of Co were synthesized by using the sol-gel method, and were fully acid washed before use. The samples were denoted as follows: Sr_2_IrCoO_x_ (SI_1_C_1_), Sr_3_Ir_2_CoO_x_ (SI_2_C_1_), Sr_5_Ir_4_CoO_x_ (SI_4_C_1_), Sr_7_Ir_6_CoO_x_ (SI_6_C_1_), Sr_9_Ir_8_CoO_x_ (SI_8_C_1_), and SrIrO_x_ (SI). The crystal structures of these samples were analyzed through high-resolution transmission electron microscopy (HRTEM) and X-ray diffraction (XRD) in Fig. 1b–h, respectively. For HRTEM testing, three samples (SI, SI_6_C_1_, and SI_1_C_1_) were selected. Specifically, SI exhibited an orthorhombic crystal structure. Fast Fourier transform (FFT) showed that the primary exposed crystal planes included (112), (110), and (002). Despite its low surface crystallinity, SI_6_C_1_ still clearly exposed crystal planes such as (112), (204), and (020). Meanwhile, SI_1_C_1_ displayed poor surface crystallinity with only a few areas showing short-range ordered crystallinity and no clear diffraction spots in the FFT pattern. To investigate the reason for the diminished crystallinity observed in SI_6_C_1_ sample, we conducted electron energy loss spectroscopy (EELS) analyses, the results of which are presented in Fig. 1e and Supplementary Fig. 3. The peaks at 532, 542, and 564 eV can be ascribed to the Ir 5d, while the peak at 536 eV is attributable to the Sr 4d^31,32^. Comparative analysis reveals that the Sr 4d peak of the SI sample is generally higher than that of the SI_6_C_1_ sample, suggesting that the presence of Sr contributes to maintaining the crystalline structure. Moreover, we identified a significant discrepancy between the surface and bulk Sr concentrations within the SI and SI_6_C_1_ samples, with this effect being particularly pronounced in the SI_6_C_1_ sample. This indicates that Co dissolution also impacts the proportion of Sr presenting on the surface. Further investigations were undertaken through energy-dispersive X-ray spectroscopy (EDS) mapping (Fig. 1f). The results confirm that the dissolution of Sr/Co and the subsequent formation of IrO_x_.

XRD revealed that the diffraction peaks of SI were consistent with typical pseudocubic (Pnma) perovskites. Also, Co-doped SI also demonstrated similar diffraction peaks, confirming the successful synthesis of the perovskite-based catalyst^21,26^. As shown in Fig. 1h, Co doping significantly reduced the crystallinity of SI and resulted in the diffraction peak shifting to the high angle, indicating lattice contraction in the Co-doped SI. In particular, SI_1_C_1_ and SI_2_C_1_ displayed almost no diffraction peaks, possibly due to the substantial dissolution of surface Co and Sr by acid washing process. These findings suggest that excessive Co doping may disrupt the pseudocubic perovskite structure after acid washing, a conclusion in line with the HRTEM results. The overall composition of the samples was analyzed by using ICP-MS, as shown in Fig. 1i. Notably, there was a significant discrepancy between the Co and Ir initial ratio and the final composition ratio of the samples. Samples with poor crystallinity, specifically SI_1_C_1_ and SI_2_C_1_, exhibited the highest Co/Ir ratio, with SI_2_C_1_ slightly higher than SI_1_C_1_. This could possibly be attributed to the difficulty in maintaining the perovskite structure in SI_1_C_1_, which led to a large amount of Co dissolution during the acid washing process and, consequently, a reduced Co/Ir ratio. Despite possessing the highest Co/Ir ratios, these two samples were still significantly lower than the initial ratio, further confirming the instability of surface Co in acidic conditions. The Co/Ir ratios of SI_4_C_1_, SI_6_C_1_, and SI_8_C_1_ showed a decreasing trend from SI_4_C_1_, SI_6_C_1_ to SI_8_C_1_ as shown in. Figure 1i suggesting that lattice maintenance assists in stabilizing Co atoms in the bulk phase. Among various SI-based samples, the SI_1_C_1_ and SI_2_C_1_ displayed the lowest Sr/Ir ratio, and the Sr/Ir ratio significantly increased with the decrease of Co doping. The Sr/Ir ratio of the SI sample was slightly higher than 1, indicating a slightly higher Sr proportion compared to Ir in bulk structure, as suggested by the EELS results shown in Supplementary Fig. 3. These conclusions were further confirmed by additional characterization methods such as scanning electron microscope (SEM), X-ray photoelectron spectroscopy (XPS), and Raman spectroscopy, as shown in Supplementary Fig. 5a, b.

### High OER catalytic performance of Co-doped SrIrO_3_

**Fig. 2:**
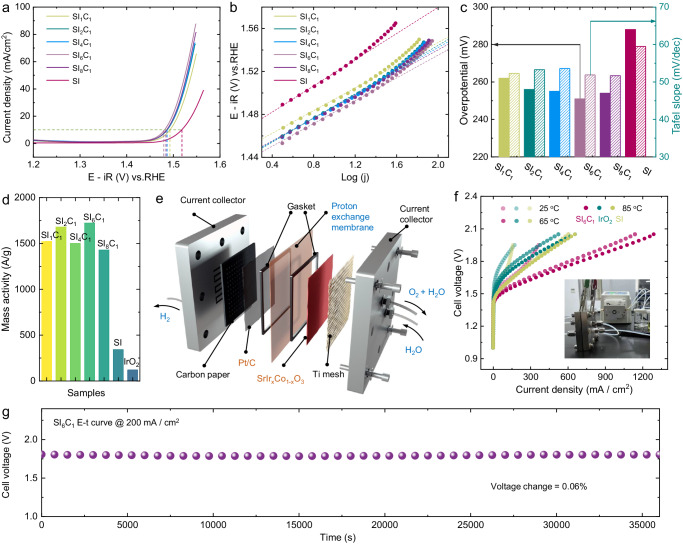
Electrochemical measurements of samples. **a** OER polarization curves of SI_1_C_1_, SI_2_C_1_, SI_4_C_1_, SI_6_C_1_, SI_8_C_1_, and SI samples with a mass loading of 0.025 mg/cm^2^ in 0.5 M H_2_SO_4_ and (**b**) corresponding Tafel slopes. The resistance values for SI_1_C_1_, SI_2_C_1_, SI_4_C_1_, SI_6_C_1_, SI_8_C_1_, SI and IrO_2_ were 3.9, 3.7, 3.6, 4.7, 3.2, 4.6 and 4.4 Ω, respectively. **c** Comparison of overvoltages and Tafel slopes of SI_1_C_1_, SI_2_C_1_, SI_4_C_1_, SI_6_C_1_, SI_8_C_1_, and SI samples. **d** Comparison of OER mass activity of SI_1_C_1_, SI_2_C_1_, SI_4_C_1_, SI_6_C_1_, SI_8_C_1_, SI and IrO_2_ samples. **e** Schematic diagram of PEM water electrolysis device. **f** PEM water electrolysis performance of SI_6_C_1_, IrO_2_, and SI samples, in set: PEM water electrolysis device photograph. **g** PEM water electrolysis stability of SI_6_C_1_ sample.

The OER catalytic performances of the SI_1_C_1_, SI_2_C_1_, SI_4_C_1_, SI_6_C_1_, SI_8_C_1_, and SI samples were investigated in acidic medium. The linear sweep voltammetry (LSV) curves for SI_1_C_1_, SI_2_C_1_, SI_4_C_1_, SI_6_C_1_, SI_8_C_1_, and SI samples were recorded after 10 cyclic voltammetry (CV) cycles, as shown in Fig. 2a. The results indicate that SI_6_C_1_ necessitates an overpotential of only 245 mV to reach a current density of 10 mA/cm^2^, which is approximately 5 times higher than that of SI at the same potential. Given that the OER catalytic process primarily occurs on the surface, the performance of SI_1_C_1_, SI_2_C_1_, SI_4_C_1_, SI_6_C_1_ and SI_8_C_1_ samples is generally superior and exhibits a similar trend. This aligns with previous characterization results, suggesting that despite varying Co doping ratios, the catalysts’ surface structures are comparable. As presented in Fig. 2b, c, the SI_1_C_1_, SI_2_C_1_, SI_4_C_1_, SI_6_C_1_ and SI_8_C_1_ samples show Tafel slopes within the range 51.5–53.6 mV/dec, significantly lower than that of SI (59.5 mV/dec). This suggests that Co participation can enhance the reaction kinetics of OER. As shown in Fig. 2d, a comparison of the mass activity of the SI_1_C_1_, SI_2_C_1_, SI_4_C_1_, SI_6_C_1_, SI_8_C_1_, SI and IrO_2_ samples reveals that the Co-doped samples all demonstrated high activity, significantly surpassing those of SI and IrO_2_.

To study the potential applications of SI series catalysts in water electrolysis, we further examined the electrocatalytic performance of three samples (SI, SI_6_C_1_, and IrO_2_) for water electrolysis by PEM. The schematic diagram of the PEM water electrolyzer is presented in Fig. 2e. The performance results of the PEM water electrolysis, shown in Fig. 2f, reveal that the catalytic activity of SI_6_C_1_ is significantly higher than those of SI and IrO_2_. It can achieve a current density exceeding 1000 mA/cm^2^ at 2.0 V cell voltage at 85 °C, which is much higher than those of the SI catalyst (580 mA/cm^2^) and IrO_2_ catalyst (560 mA/cm^2^) under the same conditions. We further tested the long-term stability of SI_6_C_1_ catalyst for PEM water electrolysis as shown in Fig. 2g and Supplementary Fig. 6. The results indicate that the SI_6_C_1_ catalyst exhibits high stability, with a performance decay rate of 0.21 mV/h, which is comparable to that of the SI catalyst and commercial IrO_2_ catalyst. This exceptional high stability indicates that the SI_6_C_1_ catalyst exhibits the potential for practical application in PEM water electrolysis.

### The roles of Co doping and dissolution in SrIrO_3_ studied by theoretical calculations

**Fig. 3:**
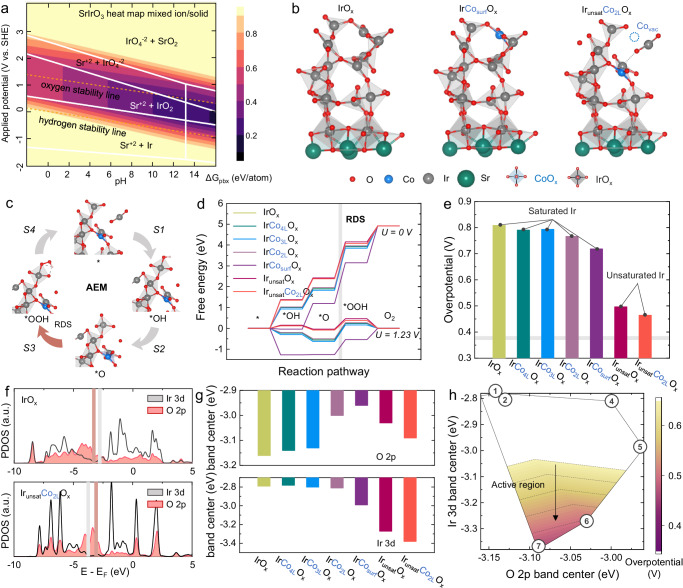
Theoretical calculations of SrIrO_3_-based perovskite catalysts. **a** Pourbaix diagram of SrIrO_3_. **b** Possible computational models of IrO_x_, IrCo_surf_O_x_ (surface Co doping) and Ir_unsat_Co_2L_O_x_ (second layer Co doping with unsaturated IrO_x_) for DFT calculations. **c** Adsorbate evolution mechanism (AEM) diagram. **d** OER free energy diagrams of different models. **e** Overpotential of different computational models. **f** Density of states diagrams for IrO_x_ and Ir_unsat_Co_2L_O_x_, and (**g**, **h**) energy band center and volcano plot for different computational models.

The impact of Co doping on SrIrO_3_ was investigated through a theoretical study. As numerous early studies had substantiated that SrIrO_3_-based catalysts undergo significant Sr dissolution during OER^23–25^. This was examined by analyzing the Pourbaix diagrams of SrIrO_3_ and Sr_4_Ir_3_CoO_12_, particularly focusing on their stability in acidic medium (Fig. 3a and Supplementary Fig. 7). The findings reveal a marked similarity in the stability properties of SrIrO_3_ and Sr_4_Ir_3_CoO_12_. Under acidic conditions, Sr displays a thermodynamic inclination towards dissolution, thereby exposing a multitude of IrO_x_ sites on the surface. Additionally, Co in the Sr_4_Ir_3_CoO_12_ also exhibits instability in acidic medium, which could also result in substantial dissolution, consistent with the EDS mapping and ICP-MS results in Fig. 1.

As indicated by the Pourbaix diagram, the structure of catalyst is significantly influenced by the applied voltage. However, the two catalysts do not exhibit notable thermodynamic differences near the oxygen stability curve, although they display pronounced differences in thermodynamic tendencies across varying pH values. This suggests that the effect of SrIrO_3_ dissolution at different OER potentials may be significantly less than the effect of electrolyte pH, particularly in strongly acidic medium. Previous research suggests that the Ir-O coordination number of surface IrO_x_ on SrIrO_3_ during OER catalysis is ~4.5^25^. While the presence Sr content at the trace amount has been detected in numerous studies, determining the exact state of Sr at the subnanometer scale remains a challenge^23^. By combining the results from the Pourbaix diagrams and above characterizations, seven models were constructed to investigate the theoretical OER catalytic sites and catalytic performance of SrIrO_3_ and Co-doped SrIrO_3_ (Fig. 3b and Supplementary Fig. 8).

The theoretical calculations were conducted on the models, as shown in Fig. 3c. The computed free energy diagrams (Fig. 3d) reveal that the rate-determining step for all models is the formation of *OOH. The Co doping, whether at the surface and bulk phase, which would not change the coordination structure of IrO_6_ octahedron, can only enhance OER catalytic activity to a certain extent. However, such activity enhancements are restricted, especially for bulk-doped Co, where the OER overpotential is reduced by only 15 ~ 42 mV. In contrast, the surface unsaturated IrO_x_, which forms following Co dissolution, exhibits a significant improvement in the catalytic activity for oxygen evolution, with the overpotential decreasing by 311 ~ 344 mV (Fig. 3e). These findings suggest that Co may not directly participate in the catalysis, but rather promote the surface reconstruction through site dissolution, leading to rapid exposure of more low-coordination IrO_x_ active sites.

To further investigate the activity origin, density of states (DOS) analyses were performed on these models (Fig. 3f). The data suggest that Co doping significantly affects the Co-O-Ir bridge oxygen, shifting the O 2*p* band center closer to the Fermi level. According to the related studies, such a shift in the O 2*p* band center substantially affect the surface stability of the catalyst and may be associated with oxygen dissolution^33,34^. This indicates that the primary effect of Co doping is to alter the surface stability of SrIrO_3_, and promote the surface reconstruction and the formation of IrO_x_ active sites. Additionally, the doping and dissolution of Co significantly influence the Ir 3*d* band center (Fig. 3g). The displacement of the metal 3*d* orbitals typically directly impacts the binding energy to the adsorbate. In this study, the negative shift of the Ir 3*d* band center led to a decrease in *OOH free energy, significantly enhancing the OER catalytic activity (Fig. 3g). By establishing the relationship between the Ir 3*d* and O 2*p* band centers and the theoretical overpotential, a new OER volcano plot was formulated (Fig. 3h). The data revealed that the moderate O 2*p* band center and the lower Ir 3*d* band center play crucial roles in the catalytic activity of SrIrO_3_-based catalysts.

### In situ characterizations of surface structural changes of catalysts

**Fig. 4:**
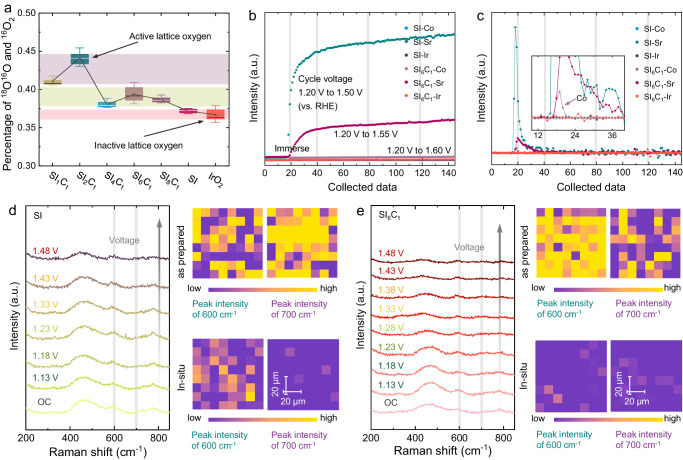
In situ characterizations of samples. **a** The ^18^O^16^O percentage of the samples test by DEMS. **b** In situ ICP-MS diagram of SI and SI_6_C_1_ and (**c**) its differential transformation diagram, in set: enlarged diagram. **d**, **e** In situ Raman and Raman mapping of SI samples and SI_6_C_1_ samples.

To elucidate the catalytic mechanism, DEMS tests were first conducted, as shown in Fig. 4a. The surfaces of SI_1_C_1_, SI_2_C_1_, SI_4_C_1_, SI_6_C_1_, SI_8_C_1_, SI, and IrO_2_ samples were labeled with ^18^O, as shown in Supplementary Figs. 10–12. For each sample, *m*/*z* = 34 signals at different cyclic voltammetry cycles were collected, and the ratio of ^18^O^16^O to ^16^O_2_ was utilized to eliminate the natural abundance of ^18^O in the air. As shown in Supplementary Fig. 13, the lattice oxygen evolution reaction (LOER) trend of most catalysts across different cyclic voltammetry cycles is similar, and the Co doping ratio appears to facilitate the release of lattice oxygen. The results indicated that Co doping reduces the stability of the surface oxygen in SrIrO_3_, consistent with O 2*p* center calculation results_._ Notably, the LOER of SI_1_C_1_ and SI_2_C_1_ is the most prominent, with SI_2_C_1_ slightly surpassing SI_1_C_1_. This phenomenon has been previously explained by XRD and ICP-MS characterizations, as SI_1_C_1_ has difficulty in maintaining the perovskite lattice, the proportion of Co doping decreases. In contrast, SI and IrO_2_ exhibit the smallest LOER process, which is linked to the stability of the catalyst surface lattice oxygen. The results indicated that Co doping and dissolution can activate the catalyst lattice oxygen, thereby accelerating the formation of IrO_x_.

In addition to LOER, in situ ICP-MS experiments were performed to detect the phenomenon of ion dissolution during OER, as shown in Fig. 4b, c. We observed that both SI_6_C_1_ and SI undergo considerable dissolution when immersed in an acidic electrolyte. Coupling this observation with prior XRD and Raman results, it suggests that the dissolution process is triggered by the rapid dissolution of Sr/Co-related oxides or compound heterophases. Furthermore, the subsequent steady dissolution trend indicates that ion dissolution of the catalyst does not necessarily occur during the catalytic process. It is noteworthy that the Sr dissolution phenomenon of SI is more significant than that of SI_6_C_1_, which is attributed to the higher concentration of surface and bulk Sr in SI. Moreover, SI_6_C_1_ is also accompanied by a small amount of Co dissolution, thus further confirming that Co dissolution promotes the formation of unsaturated IrO_x_.

To confirm the structural information of the catalyst, an in situ Raman mapping study was conducted, as shown in Fig. 4d, e. The peak around 600 cm^−1^ can be attributed to the Ir-*μ*-oxo stretching vibration of IrO_x_ (involving the unprotonated bridge oxygen, Ir^3+^), and the characteristic peaks around 550 cm^−1^ and 720 cm^−1^ can be attributed to the typical E_g_ and B_2g_ vibrational peaks of IrO_2_. In addition, the characteristic peaks around 300 and 700 cm^−1^ can be attributed to Sr-related oxide or compound^35–38^. The figure shows that before and after the OER process, SI exhibits an obvious Ir-*μ*-oxo stretching vibration peak at 600 cm^−1^, confirming the existence of the perovskite IrO_6_ structure. However, for SI_6_C_1_, a significant decrease in the Ir-*μ*-oxo stretching vibration peak is observed. This may be attributed to the destruction of the IrO_6_ structure by Codissolution to form the low-coordination IrO_x_ structure, leading to a significant reduction in peak intensity. Moreover, the characteristic peaks of Sr-related oxides/compounds near 300 and 700 cm^−1^ nearly disappear from the open circuit voltage (OC). Together with the results of in situ ICP–MS, it can be further confirmed to be caused by the dissolution of Sr-related oxides/compounds.

### Key evidences of highly active low-coordination IrO_x_ in Co-doped SrIrO_3_ for OER

**Fig. 5:**
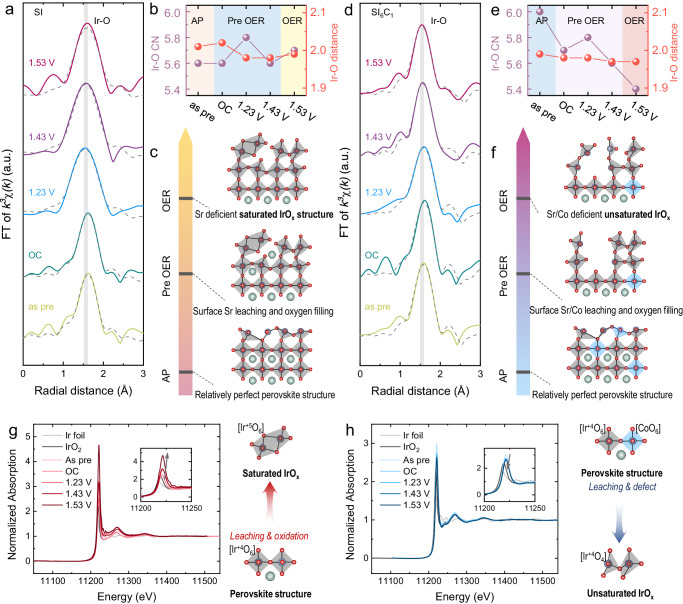
In situ XAS characterization of samples. **a** R-space fitting diagram for SI sample. **b** Changes in Ir-O coordination and Ir-O bond length of SI sample. **c** Structure change diagram of SI sample; **d** R-space fitting diagram of SI_6_C_1_ sample. **e** Ir-O coordination and Ir-O bond length variation diagram of SI_6_C_1_ sample. **f** Structure change diagram of SI_6_C_1_ sample. **g** Ir–*L*_III_ Absorption edge diagram of SI sample, in set: enlarged diagram. **h** Ir–*L*_III_ absorption edge diagram of SI_6_C_1_ sample, in set: enlarged diagram.

To elucidate the formation mechanism of IrO_x_ on the surface, comprehensive in situ EXAFS spectra was recorded at the Ir-*L*_III_ edge to monitor the evolution of local coordination of Ir, as shown in Fig. 5a–e. Initially, we examined the coordination changes of the SI sample, as demonstrated in Fig. 5a, b. The pristine state SI sample owns a Ir-O coordination number of 5.6, indicating the existence of substantial intact Ir-O octahedral structure within the catalyst. During the pre-OER stage (OC, 1.23 V, 1.43 V vs. RHE), the Ir-O coordination numbers of SI sample exhibited a pattern of initial increase followed by a decrease.

Previous reports showed a significant augmentation in the Ir-O coordination number of SrIrO_3_ thin film samples during OER, which was ascribed to the oxygen refilling process^24,25^. In this study, the dissolution of Sr led to alterations in the surface structure of SI. However, there was no significant LOER in SI sample. Therefore, during the OER oxidation process, the rate of H_2_O filling exceeded the rate of LOER, resulting in the observed trend in coordination numbers. During the OER stage (1.63 V vs. RHE), the Ir-O coordination number of the SI sample exhibited a minor increase, suggesting that at high potentials, the lattice oxygen of SI remained inactive, leading to the higher H_2_O filling rate than the LOER rate^25^. Additionally, at 1.23 V vs. RHE, a significant reduction in the Ir-O bond length of the SI sample was observed, potentially indicating a transformation of the octahedral corner-sharing structure during the H_2_O filling process^26^. In sum, as shown in Fig. 5c, the surface site mechanism of the SI sample undergoes a dynamic process from a relatively complete perovskite structure to site dissolution and adsorbate filling, ultimately transitioning into a saturated IrO_x_ structure.

We next analyzed the coordination changes in the SI_6_C_1_ sample, as illustrated in Fig. 5d, e. The initial Ir-O coordination number of SI_6_C_1_ was 6.0, suggesting a more perfect Ir-O octahedral structure compared with SI. However, prior to the OER stage (OC, 1.23 V, 1.43 V vs. RHE), the Ir-O coordination number of the SI_6_C_1_ sample exhibited a notable decreasing trend. This is consistent with the formation of low-coordination IrO_x_ as observed in in situ ICP-MS results. Furthermore, during the OER stage, the Ir-O coordination number of SI_6_C_1_ further decreased to 5.4, significantly lower than SI, confirming that the active lattice oxygen in SI_6_C_1_ further facilitated the formation of highly active low-coordination IrO_x_.

To validate the generation of the unsaturated IrO_x_ structure, in situ XANES spectra were analyzed at the Ir-*L*_III_ edge to study the oxidation state of Ir, as shown in Fig. 5g, h. The Ir-*L*_III_ absorption edge of SI displayed an increasing white line intensity during the OER process, indicating an increase in the oxidation state of Ir. Differently, the Ir-*L*_III_ absorption edge of SI_6_C_1_ exhibited a slight decrease in white line intensity, signifying a reduction in the oxidation state of Ir. Combined with the valence state information of reference samples Ir and IrO_2_, the mechanism of Ir valence state change during OER can be inferred. During the OER process of SI, the dissolution of Sr and the filling of H_2_O led to the saturation of the Ir-O coordination, displaying a higher valence state. Conversely, for SI_6_C_1_, the dissolution of Co resulted in the formation of unsaturated Ir-O, leading to a decrease in the average Ir valence state. This observation further substantiates that Co can enhance the generation of unsaturated IrO_x_ structures, aligning with the results from prior characterizations.

## Discussion

### OER catalytic mechanism on Co-doped SrIrO_3_ catalyst

**Fig. 6:**
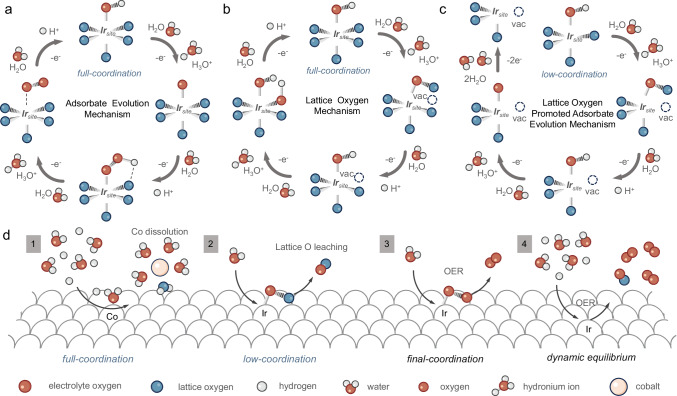
Proposed possible OER mechanism. **a** Adsorbate evolution mechanism. **b** Lattice oxygen mechanism. **c** Lattice oxygen promoted adsorbate evolution mechanism. **d** Catalytic mechanism of Co-doped SrIrO_3_ catalyst.

Our study has elucidated the surface reconstruction process of SIC series catalysts by theoretical calculations and a comprehensive series of in situ characterizations. We now proceed to discuss and summarize the potential OER mechanisms inherent to SIC series catalysts. There are two widely recognized mechanisms for OER, specifically the adsorbate evolution mechanism (AEM) and the lattice oxygen mechanism (LOM), as shown in Fig. 6a, b^39,40^. Our theoretical calculations have demonstrated that the surface O 2*p* band center of the Co/Ir model is closer to the Fermi level compared to the Ir model, which suggests that the bridging oxygen of Co-O-Ir is thermodynamically predisposed to be oxidized^40^. In situ Raman spectroscopy revealed that the Ir-*μ*-oxo stretching vibration peak (indicative of bridging oxygen) in the Co/Ir system catalyst was notably reduced during the OC and OER processes when compared to the Ir system. Further, DEMS tests have found that a higher content of Co promotes the LOM.

Despite these findings, our DEMS results suggest that the AEM remains the dominant mechanism, while the LOM significantly contributes to the formation of unsaturated IrO_x_. Consequently, we propose a distinctive catalytic mechanism, the lattice oxygen promoted adsorbate evolution mechanism (LOPAEM), as illustrated in Fig. 6c. Unlike the conventional catalytic mechanisms of OER, LOPAEM involves the synergistic action of both mechanisms. Specifically, when the oxidation rate of lattice oxygen in certain catalysts becomes excessively fast, it will lead to the formation of a large number of surface oxygen vacancies (i.e., unsaturated metal sites), thereby altering the original catalytic sites of the catalyst. These unsaturated metal sites may exhibit more efficient AEM performance, thus achieving LOPAEM. Our findings suggest that the LOM in Co/Ir system catalysts is an integral step for catalyst activation. The formation of Ir-O-Co_vac_ substantially reduces the catalyst’s AEM overpotential, thereby making it thermodynamically more favorable. This is in agreement with our theoretical calculations and DEMS results. Moreover, the LOM process is unable to produce a lower Ir-O coordination structure as the dissolution of lattice oxygen shifts the O 2*p* center away from the Fermi level, precluding further oxidation (as detailed in the theoretical calculation section).

In light of the above findings, we have summarized the mechanism for the Co/Ir system catalyst as represented in Fig. 6d. Initially, Co on the catalyst’s surface dissolves under acidic conditions, removing a portion of the bridging lattice oxygen to form an IrO_x_ structure with lower coordination. Subsequently, lattice oxygen dissolves during the OER process, forming an unsaturated IrO_x_ structure that exhibits high AEM activity. Lastly, under the dynamic balance of adsorbate filling and LOM, the catalyst conducts AEM to efficiently facilitate the OER.

In summary, we designed a highly active catalyst through Co doping and dynamic dissolution of Co/Sr bimetallic ions to study the catalytic mechanism of SrIrO_3_-based perovskite. Theoretical calculations and in situ characterizations (DEMS, in situ Raman mapping, in situ XAS and in situ ICP-MS) show that dynamic dissolution of Co is crucial for forming highly active unsaturated IrO_x_. The as-synthesized catalysts exhibit higher OER reaction kinetics than SrIrO_3_ and commercial IrO_2_ catalyst in both electrolyzer and PEM water electrolyzer, revealing the mechanism of catalytic activity enhancement by tuning catalytic sites. This work is of great significance for understanding the high OER catalytic performance of Ir-based catalysts, and will provide an important basis for the design and preparation of high-performance acidic OER catalysts.

## Methods

### Chemicals

The chemical reagents utilized in this study were all received from the manufacturer. Potassium hexachloroiridate (IV) [K_2_IrCl_6_, AR, Macklin], cobalt(III) nitrate hexahydrate [Co(NO_3_)_3_·6H_2_O, AR, Sigma-Aldrich], strontium(II) nitrate [Sr(NO_3_)_2_, AR, Guangdong chemical reagent)], citric acid monohydrate (AR, LookChem.) were utilized as precursors.

#### Preparation of SI Catalyst

Solution A was prepared by dissolving Sr(NO_3_)_2_ (280 mg) and citric acid (840 mg) in 5.0 mL of deionized water. Solution B was prepared by dissolving K_2_IrCl_6_ (80 mg) in 4.0 mL of ethylene glycol. Solution A was then added dropwise with stirring to solution B. The resulting mixture was dried at 150 °C for 12 h to obtain a brown solid product as a precursor. Subsequently, the precursor was calcined in air at 200 °C for 6 h, 300 °C for 6 h, 500 °C for 3 h, and 700 °C for 6 h with a heating rate of 2 °C/min. Afterward, the excess SrCO_3_ impurities were removed by reacting with a 1.0 M HCl solution for 12 h to obtain SrIrO_3_ (SI) catalyst.

#### Preparation of SI_1_C_1_ catalyst

Solution A was prepared by dissolving Sr(NO_3_)_2_ (280 mg) and citric acid (840 mg) in 5.0 mL of deionized water. Solution B was prepared by dissolving K_2_IrCl_6_ (40 mg) and Co(NO_3_)_2_ (24 mg) in 4.0 mL of ethylene glycol. The subsequent steps were identical to the preparation of the SI catalyst described above.

#### Preparation of SI_2_C_1_ catalyst

Solution A was prepared by dissolving Sr(NO_3_)_2_ (280 mg) and citric acid (840 mg) in 5.0 mL of deionized water. Solution B was prepared by dissolving K_2_IrCl_6_ (53 mg) and Co(NO_3_)_2_ (16 mg) in 4.0 mL of ethylene glycol. The subsequent steps were identical to the preparation of the SI catalyst described above.

#### Preparation of SI_4_C_1_ catalyst

Solution A was prepared by dissolving Sr(NO_3_)_2_ (280 mg) and citric acid (840 mg) in 5.0 mL of deionized water. Solution B was prepared by dissolving K_2_IrCl_6_ (64 mg) and Co(NO_3_)_2_ (10 mg) in 4.0 mL of ethylene glycol. The subsequent steps were identical to the preparation of the SI catalyst described above.

#### Preparation of SI_6_C_1_ catalyst

Solution A was prepared by dissolving Sr(NO_3_)_2_ (280 mg) and citric acid (840 mg) in 5.0 mL of deionized water. Solution B was prepared by dissolving K_2_IrCl_6_ (68 mg) and Co(NO_3_)_2_ (7 mg) in 4.0 mL of ethylene glycol. The subsequent steps were identical to the preparation of the SI catalyst described above.

#### Preparation of SI_8_C_1_ catalyst

Solution A was prepared by dissolving Sr(NO_3_)_2_ (280 mg) and citric acid (840 mg) in 5.0 mL of deionized water. Solution B was prepared by dissolving K_2_IrCl_6_ (71 mg) and Co(NO_3_)_2_ (5 mg) in 4.0 mL of ethylene glycol. The subsequent steps were identical to the preparation of the SI catalyst described above.

#### Materials characterizations

Characterization of the atomic-level crystal structure was performed using an aberration-corrected scanning transmission electron microscope (JEM-ARM200P, JAPAN) operated at 300 kV. Energy-dispersive X-ray (EDX) analysis was used to measure the relative elemental content. X-ray diffraction (XRD) patterns of SrIrO_3_ were recorded on an X-ray diffractometer (Smart lab) using Cu-Kα radiation (*λ* = 1.5418 Å) with a step size of 0.02° and a step time of 0.2 s in the 20°–80° range. X-ray photoelectron spectroscopy (XPS) was performed using a Thermo Scientific K-Alpha X-ray photoelectron spectrometer, and all XPS spectra were calibrated using the C 1*s* line at 284.8 eV. The surface morphology of SrIrO_3_ was characterized using a scanning electron microscope (TESCAN MIRA LMS). Considering the high acid resistance of SrIrO_3_, anti aqua regia was prepared by mixing hydrochloric acid and nitric acid in a 1:3 ratio for the experiment. In this solution, 1.0 mg of SrIrO_3_ powder was dissolved in 10 mL of aqua regia and left to stand for 1–3 week after thorough ultrasonic treatment. Finally, the proportions of each element in SrIrO_3_ were determined by ICP-MS (iCAP RQ) analysis.

#### In situ characterizations and LOER confirmation

X-ray absorption spectra of Ir *L*-edge were obtained at the BL17B and BL20U1 beamline of the Shanghai Synchrotron Radiation Facility (SSRF). The spectra were collected either in transmission mode or fluorescence mode using a Lytle detector. The corresponding reference samples were collected in transmission mode. The samples were ground and uniformly applied to special adhesive tape. In situ XANES characterizations were performed in fluorescence mode at the same beamline. The samples were sprayed onto carbon paper at a loading of 1.0 mg cm^−2^ as the working electrode. The measurements were conducted under the same conditions as the OER measurements in a self-designed cell. The in situ Raman spectroscopy characterizations were carried out using an inVia confocal Raman microscope from Renishaw. The laser power was set at 532 nm with 1% power at a grating of 1800 mm/1, the silicon peak was calibrated before testing. During the OER process, differential electrochemical mass spectrometry (DEMS) measurements were conducted using the QAS 100 apparatus from Shanghai Linglu Instruments to determine the volatile reaction products of the SI series catalysts and IrO_2_ catalyst labeled with ^18^O. Saturated Ag/AgCl and Pt wires served as the reference electrode (RE) and counter electrode (CE), respectively. The working electrode (WE) was prepared by sputtering Au onto a 50 μm-thick porous PTFE membrane, followed by depositing 10 μL of catalyst ink (1.0 mg/mL) onto the Au surface. The catalyst isotopic labeling was achieved by cycling the electrode in H_2_^18^O for eight cycles using cyclic voltammetry (CV) with a scan rate of 5 mV/s in the range of 0–0.5 V vs. Ag/AgCl. Subsequently, the ^18^O-labeled electrode was rinsed with H_2_^16^O to remove residual H_2_^18^O. Finally, the electrode was electrochemically tested against Ag/AgCl in a 1.0 M H_2_SO_4_ solution at different potentials with a scan rate of 5 mV/s. The DEMS signal was normalized by current density (A/g). Simultaneously, real-time measurements of gas products with different molecular weights generated during the OER process were conducted using mass spectrometry. In situ ICP-MS experiments were performed using a Thermo Scientific iCAP RQ instrument. The experimental setup consisted of a standard three-electrode cell with a 3 mm glassy carbon working electrode, consistent with the electrochemical testing. The reference electrode used was a saturated calomel electrode (Hg/HgCl_2_), and the counter electrode was a platinum foil electrode. To ensure accurate detection of ion distribution, a stirrer was employed to prevent leaching and dissolution errors, with data sampling occurring every 15 s.

#### Electrochemical characterizations

To prepare the catalyst ink, a 0.5 mg amount of catalyst was mixed with 1.0 ml of a 0.05 wt% Nafion solution and neutralized. Subsequently, a 10 μl volume of the prepared ink was deposited onto a glassy carbon electrode (GCE) with a diameter of 5 mm and dried using an infrared lamp. Prior to use, the GCE was polished with 0.05 μm alumina powder and rinsed three times with a mixture of high purity water and ethanol. Electrochemical measurements were conducted in a three-electrode system using an electrochemical workstation (CHI 760E). The reference electrode used was an Hg/HgCl_2_ electrode in a 0.5 M H_2_SO_4_ electrolyte, while a carbon rod served as the counter electrode. The working electrode was the GCE with the catalyst. LSV and CV was performed in 0.5 M H_2_SO_4_ solution at a scan rate of 10 mV/s. A home-made PEM water electrolysis cell with a proton exchange membrane was used to evaluate the performance of SI series catalyst. The preparation step of catalyst inks is the same as above method. The total catalyst loading on the electrode was 1.0 mg and all the catalyst inks were deposited on carbon paper (1 cm × 1 cm). The cell temperature (25, 65, and 85 °C) was maintained by an electric heating plate and measured by a temperature probe in electrolyte.

#### Computational methods

Spin-polarized density functional theory (DFT) calculations were performed in the plane wave and ultrasoft pseudopotential (USPP) with Perdew-Burke-Ernzerhof (PBE) exchange functional correction as implemented in Quantum ESPRESSO^41,42^. An energy cutoff of 25 Ry was employed for the plane wave expansion of the electronic wavefunction. The atomic structures of the models were fully relaxed until self-consistency was achieved with a convergence criteria of 10^−6^ Ry for the energy and 10^−3^ Ry/Bohr for the atomic coordinates. To prevent interaction between layers, a vacuum slab of 12 Å was used to isolate the surface. For bulk geometry optimization, a 3 × 3 × 1 Monkhorst-Pack k-point set was used, while a 5 × 5 × 1 set was used for electronic structure calculations. The correction for every adsorbate and surface, with typical values of +0.35 eV, +0.05 eV, +0.35 eV for *OH, *O and *OOH respectively. To simulate the complex unsaturated IrO_x_ structure, we start by constructing and optimizing the slab structure of the original SrIrO_3_. Then, the surface Sr atoms were removed from the SrIrO_3_ slab for further optimization. According to a previous report^25^, the Ir-O coordination number on the SrIrO_3_ surface is approximately 4.5, achieved by removing 4 Sr atoms and their corresponding 3 neighboring O atoms. The same procedure can be applied to the Co-doped system, where removing a Co atom also removes 2 neighboring O atoms.

#### XAS analysis

The acquired extended X-ray absorption fine structure (EXAFS) data were processed following standard procedures using the ATHENA module of the Demeter software package^43^. The EXAFS spectra were obtained by subtracting the post-edge background from the overall absorption and then normalized with respect to the edge-jump step. The χ(k) data were Fourier transformed to real (R) space using a Hanning window (dk = 1.0 Å^−1^) to separate the contributions from different coordination shells. To determine the quantitative structural parameters around the central atoms, least-squares curve parameter fitting was performed using the ARTEMIS module of the Demeter software package.

### Supplementary information

Supplementary Information

Peer Review File

### Source data

Source Data

## Data Availability

The data generated in this study are provided in the Supplementary Information and are available from the authors upon request. Source data are provided with this paper.
