# Supplementary material for: The formation of unsaturated IrOx in SrIrO3 by cobalt-doping for acidic oxygen evolution reaction
Source: Nat Commun. 2024 Apr 4;15:2928. doi: 10.1038/s41467-024-46801-y (PMC10995174; doi:10.1038/s41467-024-46801-y)
Supplement: Supplementary file 1 — Supplementary Information [file 41467_2024_46801_MOESM1_ESM.pdf]

Supplementary Information

The Formation of Unsaturated IrO_x in $SrIrO_3$ by Cobalt-doping for Acidic Oxygen Evolution Reaction

Jia-Wei Zhao,[1,2,3,†] Kaihang Yue,[4,†] Hong Zhang,[5,†] Shu-Yin Wei,[2] Jiawei Zhu,[3] Dongdong Wang,[3] Junze Chen,[1] Vyacheslav Yu. Fominski,[6] Gao-Ren Li[1,*]

[1]College of Materials Science and Engineering, Sichuan University, Chengdu 610065, China

[2]School of Chemistry, Sun Yat-sen University, Guangzhou 510275, China

[3]Department of Mechanical Engineering, City University of Hong Kong, 83 Tat Chee Avenue, Kowloon, Hong Kong SAR 999077, China

[4]CAS Key Laboratory of Materials for Energy Conversion, Shanghai Institute of Ceramics, Chinese Academy of Sciences (SICCAS), 585 Heshuo Road, Shanghai 200050, China

[5]Electron Microscopy Centre, School of Physical Science and Technology, Lanzhou University, Lanzhou 730099, China

[6]National Research Nuclear University MEPhI (Moscow Engineering Physics Institute), Kashirskoe sh. 31, Moscow 115409, Russia

[†]Authors contributed equally to this work

*e-mail: ligaoren@scu.edu.cn (G.-R. Li)

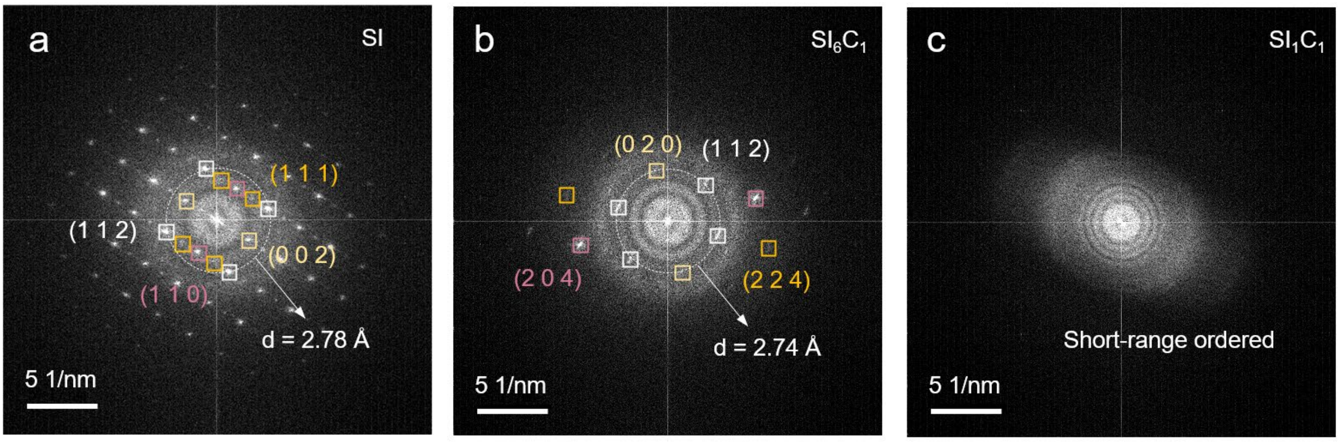

Supplementary Fig. 1 High resolution FFT diagram of (a) SI, (b) SI_6C_1 and (c) SI_1C_1.

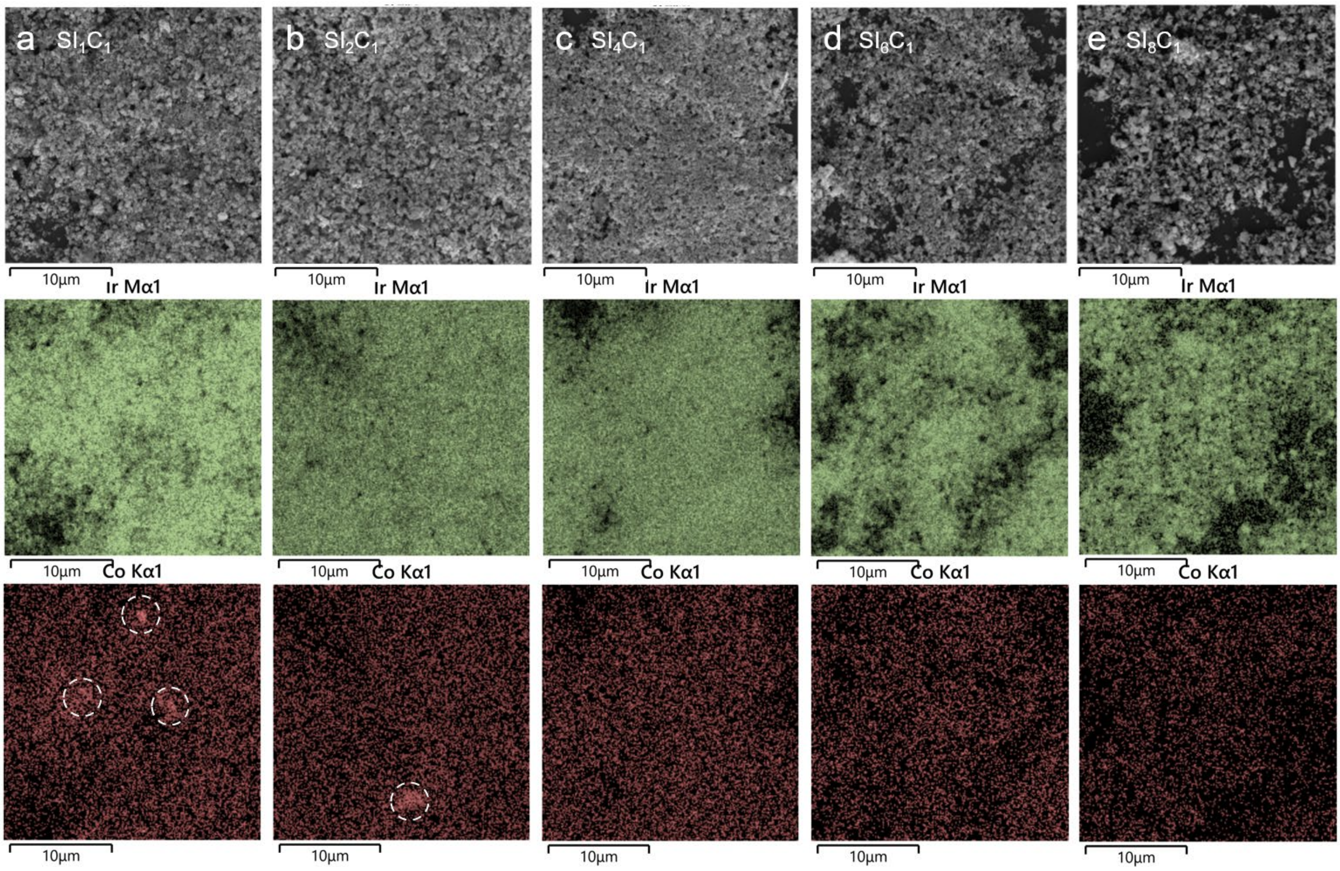

Supplementary Fig. 2 SEM images and EDS mappings of (a) SI_1C_1, (b) SI_2C_1, (c) SI_4C_1. (d) SI_6C_1 and (e) SI_8C_1.

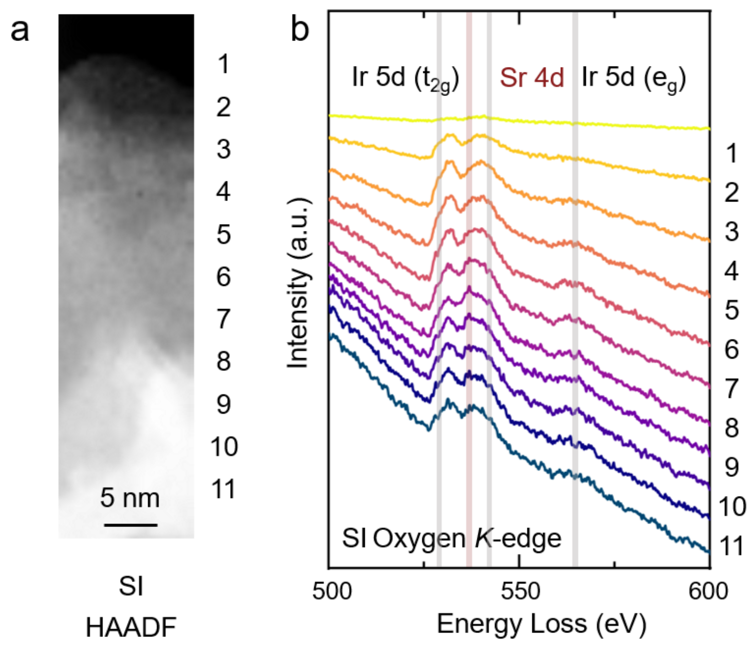

Supplementary Fig. 3 (a) HAADF image of SI; (b) O K-edge EELS spectra of SI.

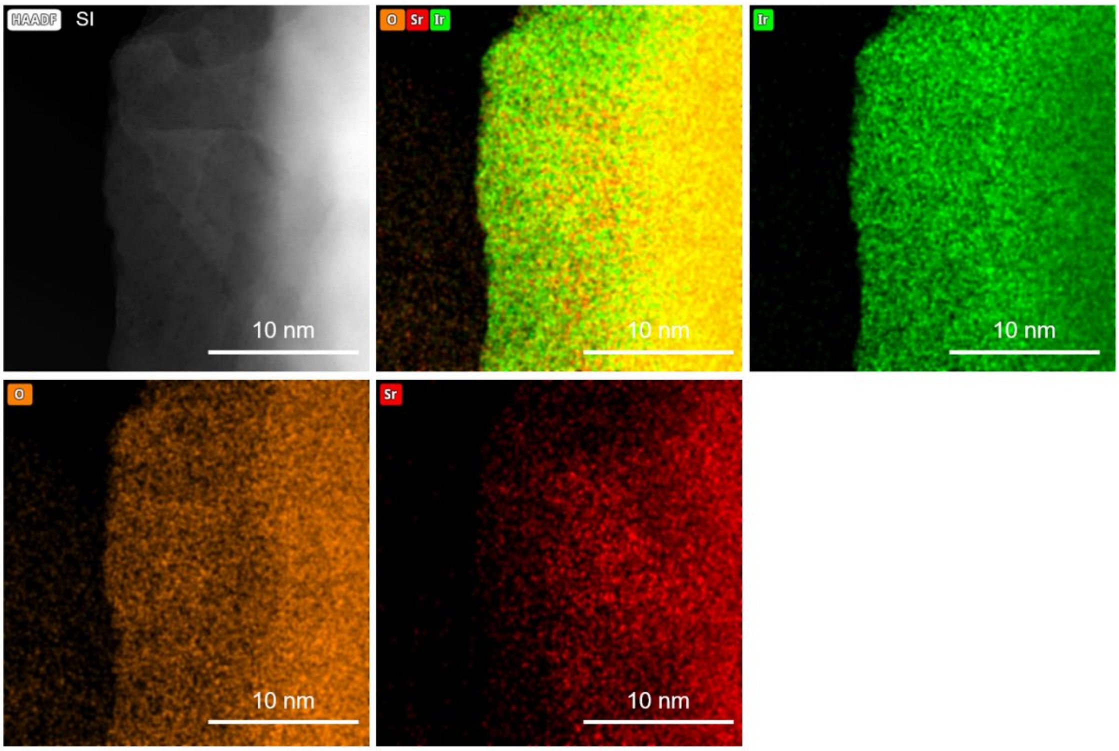

Supplementary Fig. 4 HAADF image of SI and corresponding EDS mapping images.

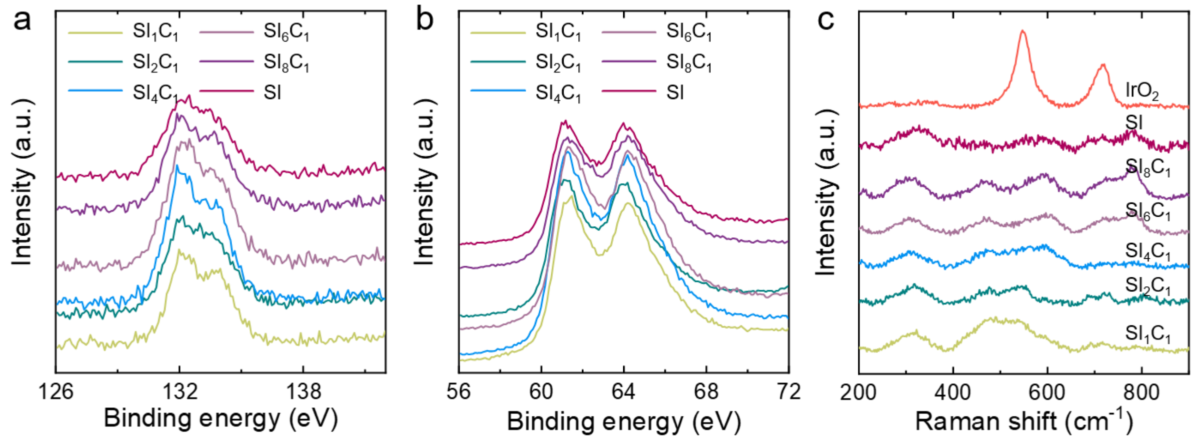

Supplementary Fig. 5 (a–b) XPS spectra of Sr *3d* and Ir *4f* for SI series samples; (c) Raman spectra of SI series samples.

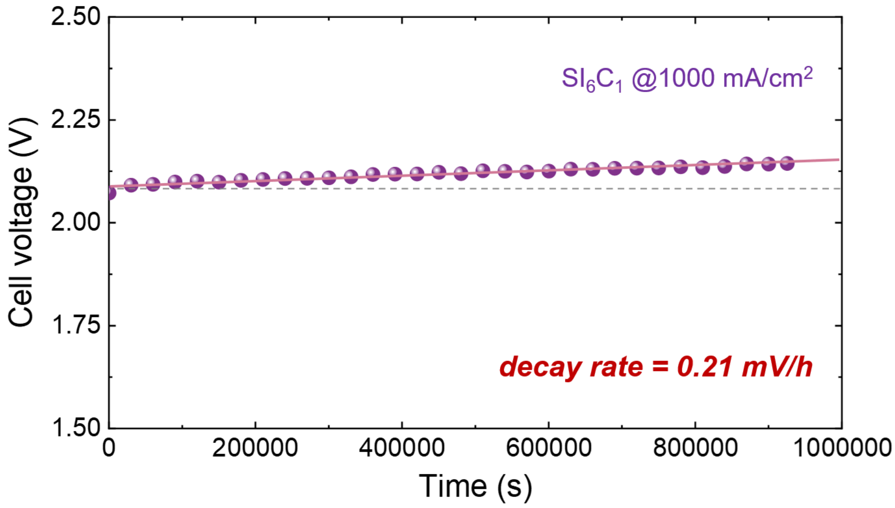

Supplementary Fig. 6 PEM water electrolysis stability of SI_6C_1 (at 1000 mA/cm^2).

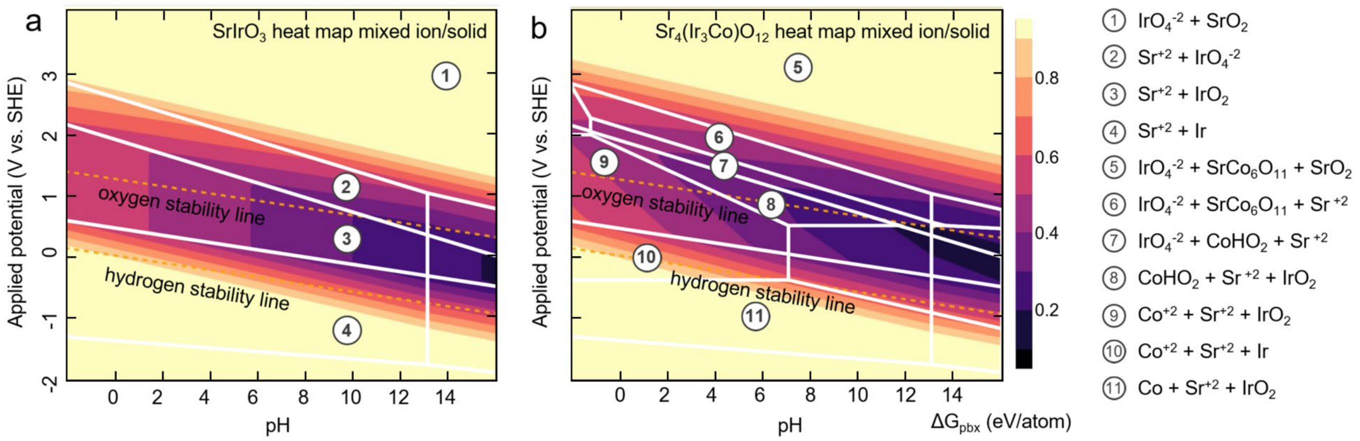

Supplementary Fig. 7 Pourbaix diagram of (a) $SrIrO_3$ and (b) $Sr_4(Ir_3Co)O_{12}$.

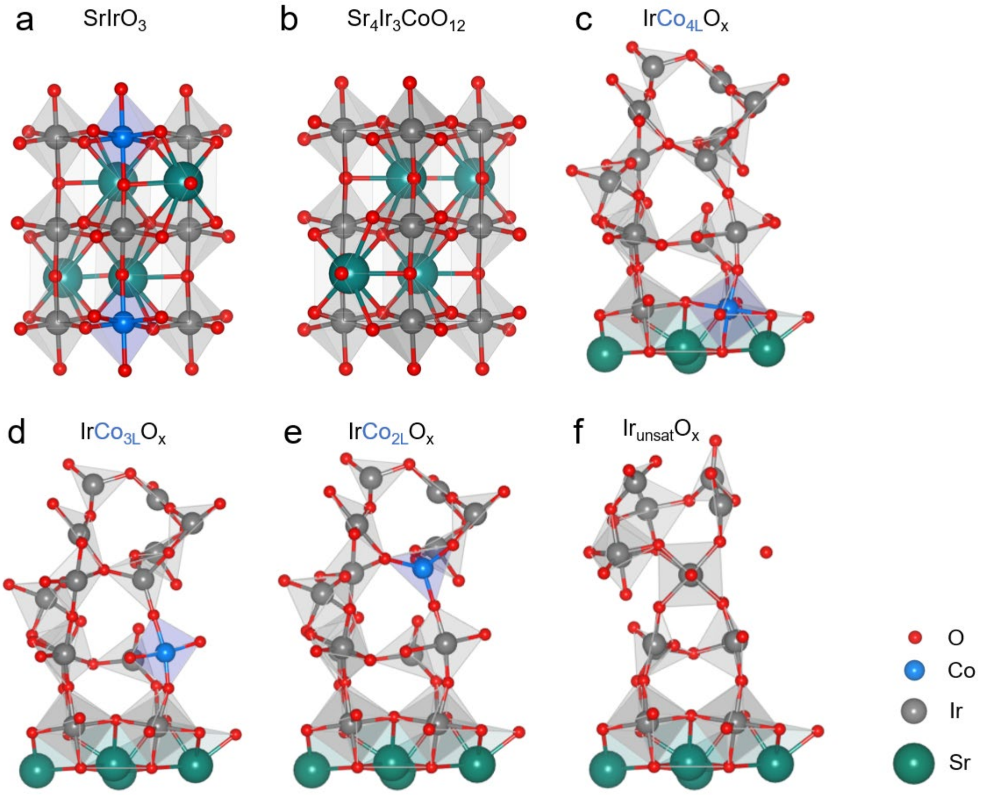

Supplementary Fig. 8 Possible computational models of (a) $SrIrO_3$, (b) $Sr_4Ir_3CoO_{12}$, (c) $IrCo_{4L}O_x$, (d) $IrCo_{3L}O_x$, (e) $IrCo_{2L}O_x$ and (f) $Ir_{unsat}O_x$ for DFT calculations.

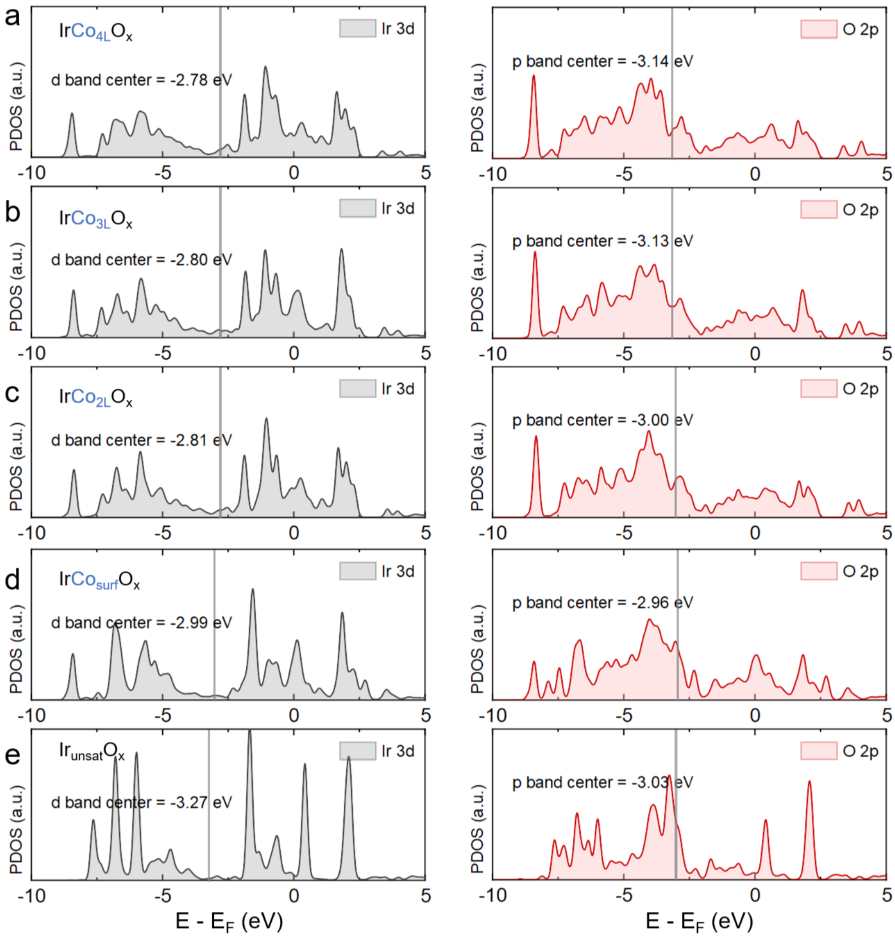

Supplementary Fig. 9 Density of states diagrams (Ir 3d and O 2p) for (a) $IrCo_{4L}O_x$, (b) $IrCo_{3L}O_x$, (c) $IrCo_{2L}O_x$, (d) $IrCo_{surf}O_x$ and (e) $Ir_{unsat}O_x$.

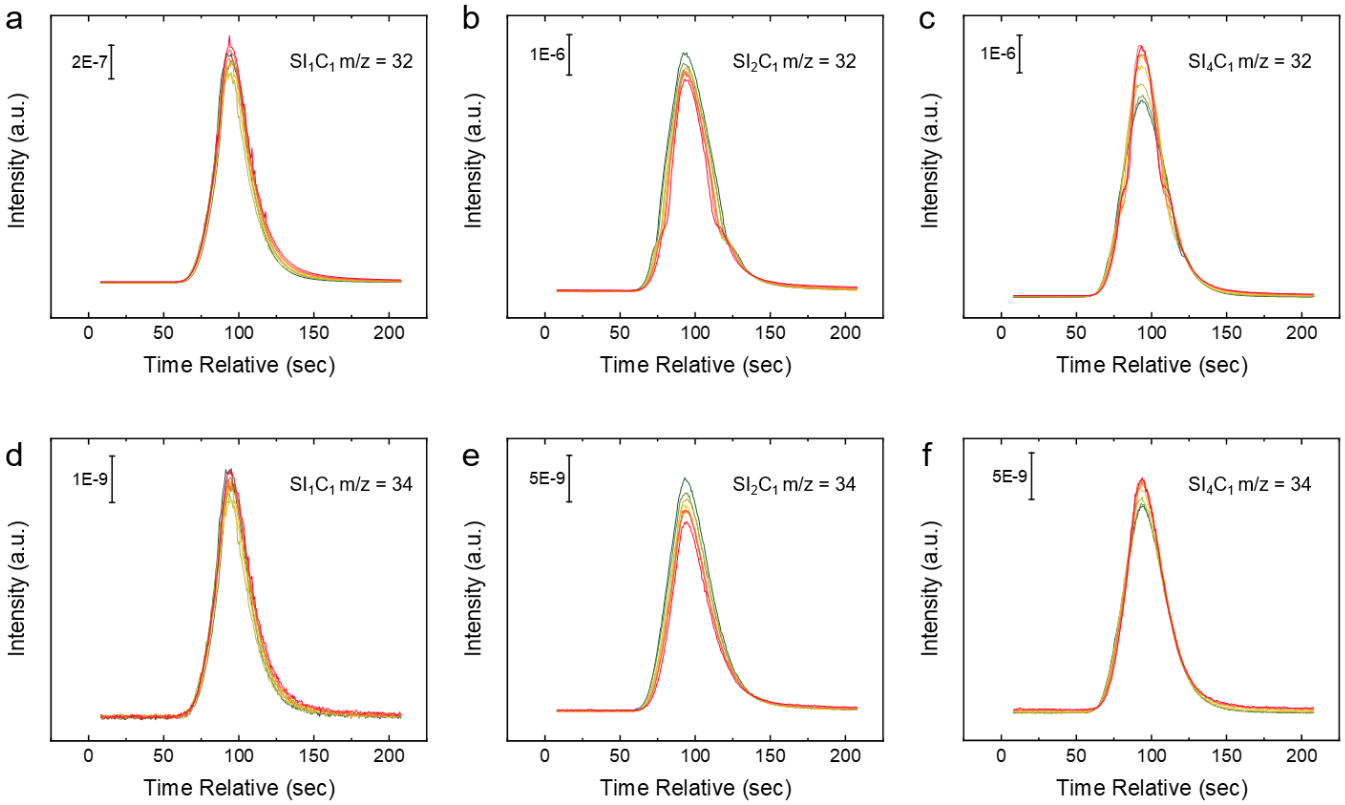

Supplementary Fig. 10 The $^{16}O^{16}O$ and $^{18}O^{16}O$ intensities of (a, d) SI_1C_1, (b, e) SI_2C_1, and (c, f) SI_4C_1 tested by DEMS.

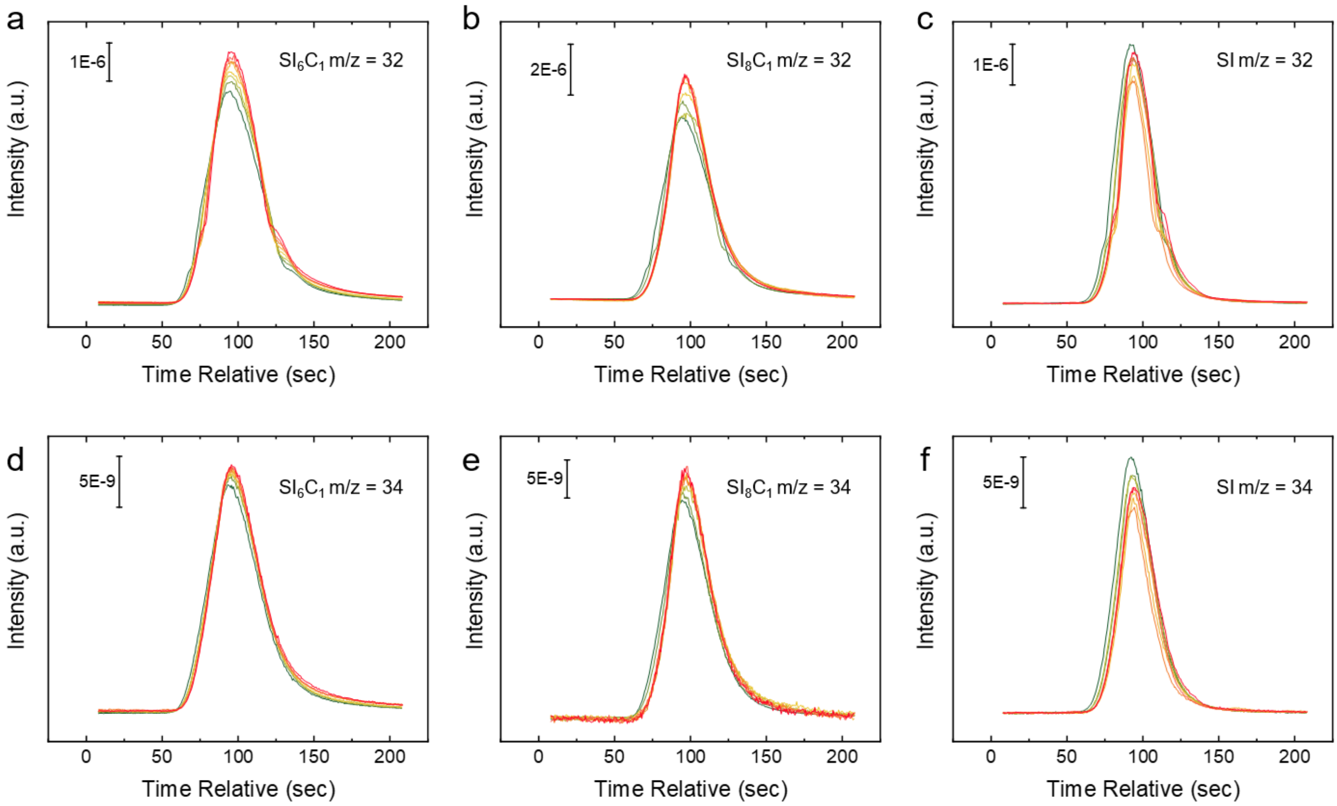

Supplementary Fig. 11 The $^{16}O^{16}O$ and $^{18}O^{16}O$ intensities of (a, d) SI_6C_1, (b, e) SI_8C_1, and (c, f) SI tested by DEMS.

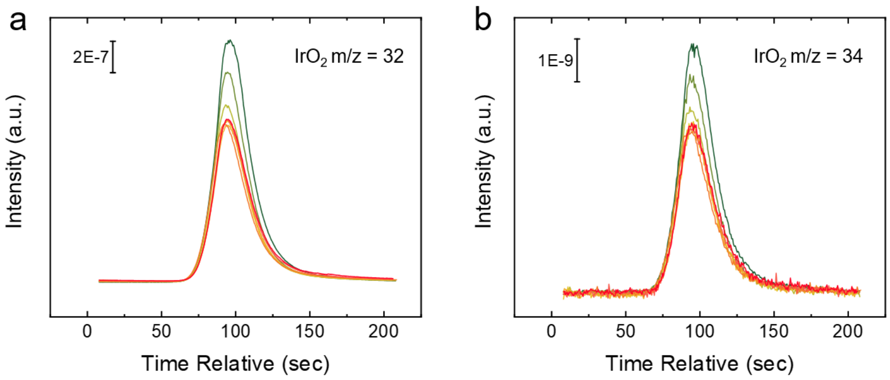

Supplementary Fig. 12 The (a) $^{16}O^{16}O$ and (b) $^{18}O^{16}O$ intensities of IrO_2 tested by DEMS.

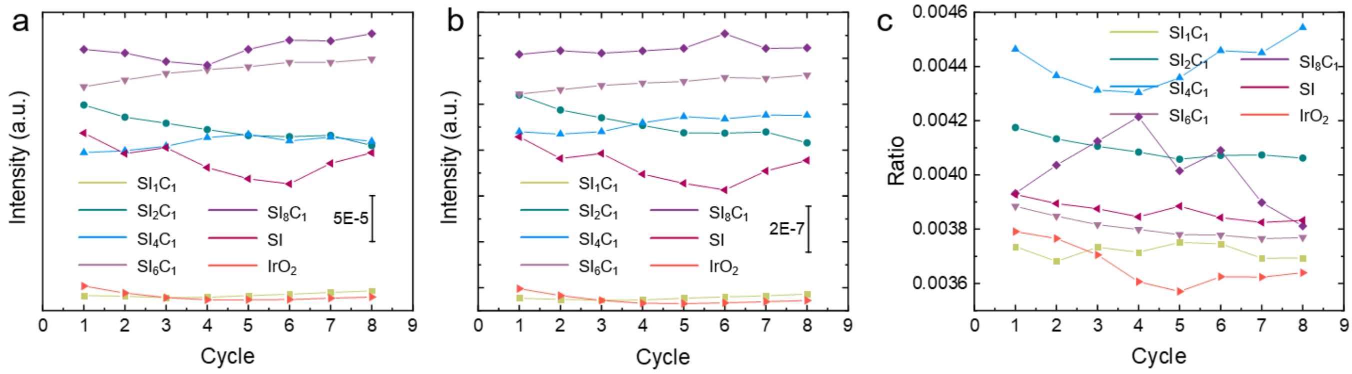

Supplementary Fig. 13 (a) The $^{32}O_2$ intensity under the different CV cycles of the samples; (b) The $^{34}O_2$ intensity under the different CV cycles of the samples; (c) The ratio diagram of $^{34}O_2$ under the different CV cycles of the samples.

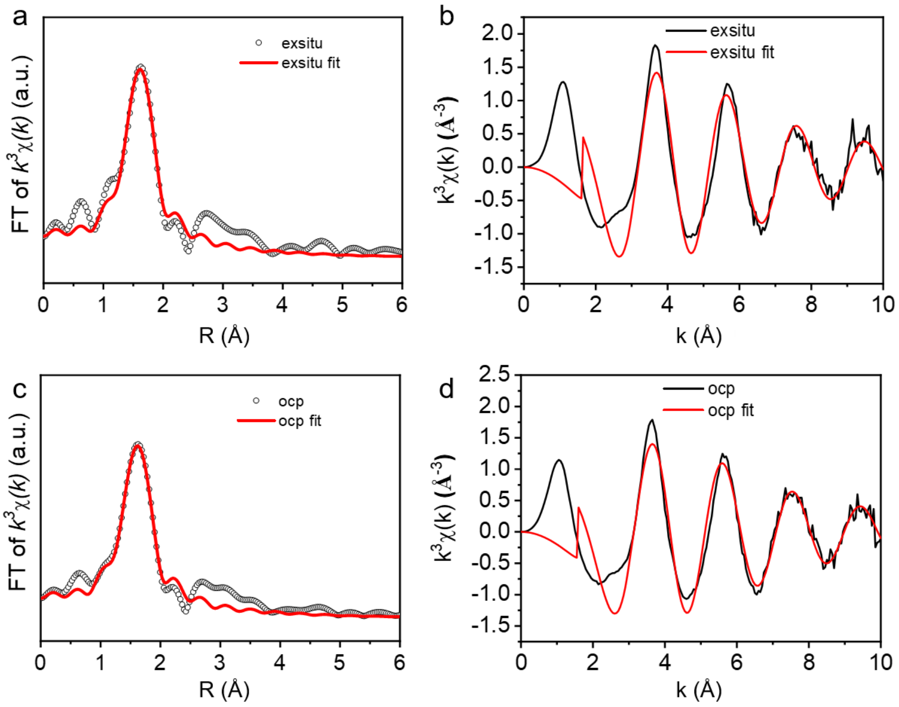

Supplementary Fig. 14 (a, c) χ(R) space spectra fitting curve of SI (ex-situ and ocp); (b, d) $k^3\chi(k)$ space spectra fitting curve of SI (ex-situ and ocp).

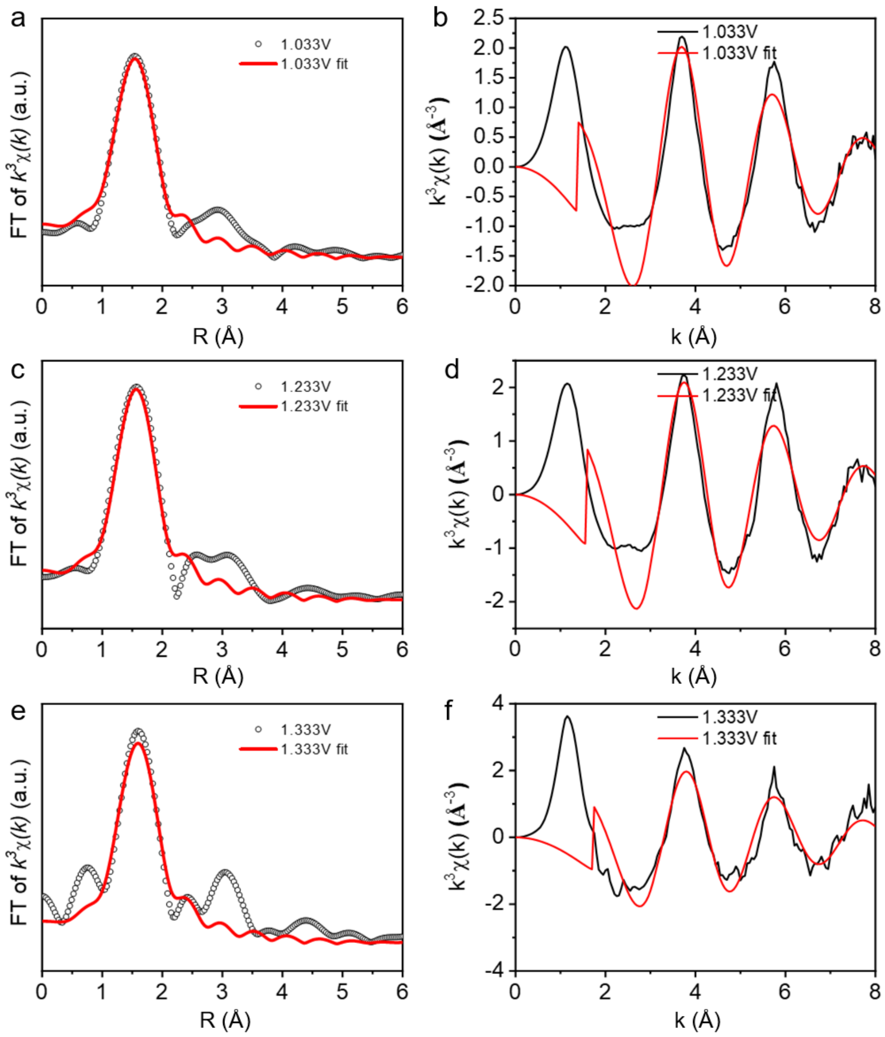

Supplementary Fig. 15 (a, c, e) χ(R) space spectra fitting curves of SI (1.033 V, 1.233 V and 1.333 V *vs*. RHE); (b, d, f) $k^3\chi(k)$ space spectra fitting curves of SI (1.033 V, 1.233 V and 1.333 V *vs*. RHE).

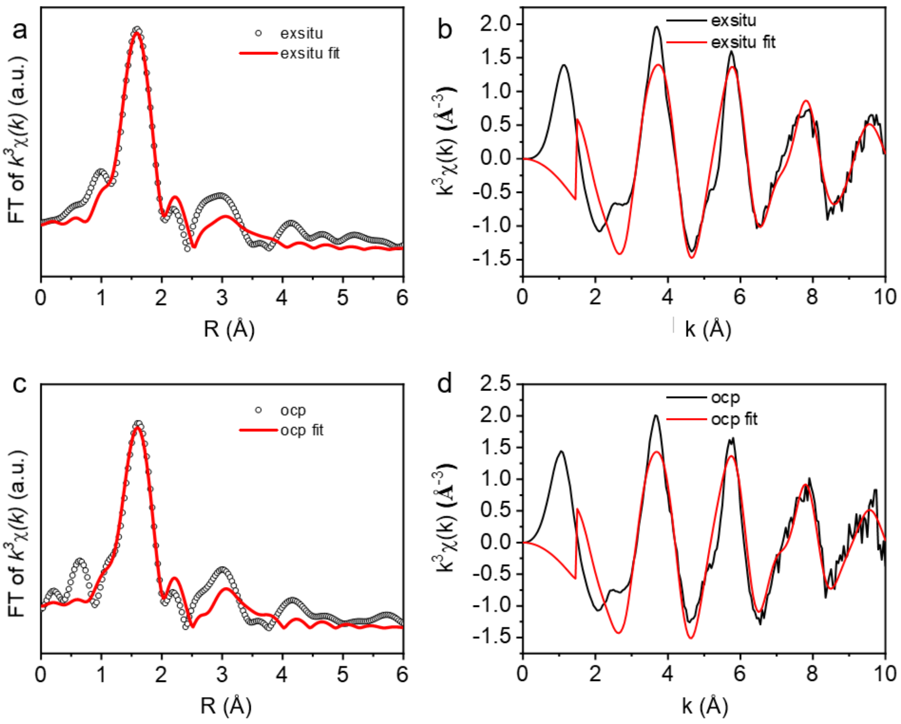

Supplementary Fig. 16 (a, c) χ(R) space spectra fitting curves of SI_6C_1 (ex-situ and ocp); (b, d) $k^3\chi(k)$ space spectra fitting curves of SI_6C_1 (ex-situ and ocp).

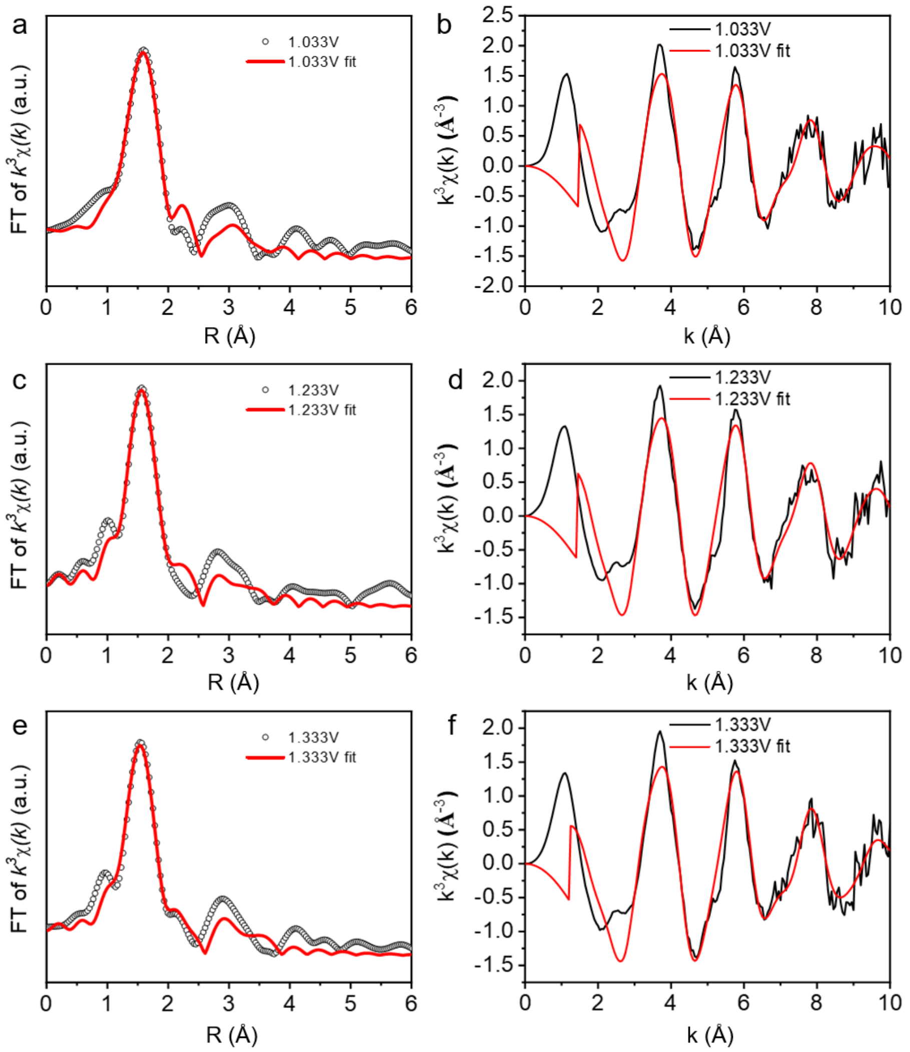

Supplementary Fig. 17 (a, c, e) χ(R) space spectra fitting curves of SI_6C_1 (1.033 V, 1.233 V and 1.333 V *vs*. RHE); (b, d, f) $k^3\chi(k)$ space spectra fitting curves of SI_6C_1 (1.033 V, 1.233 V and 1.333 V *vs*. RHE).

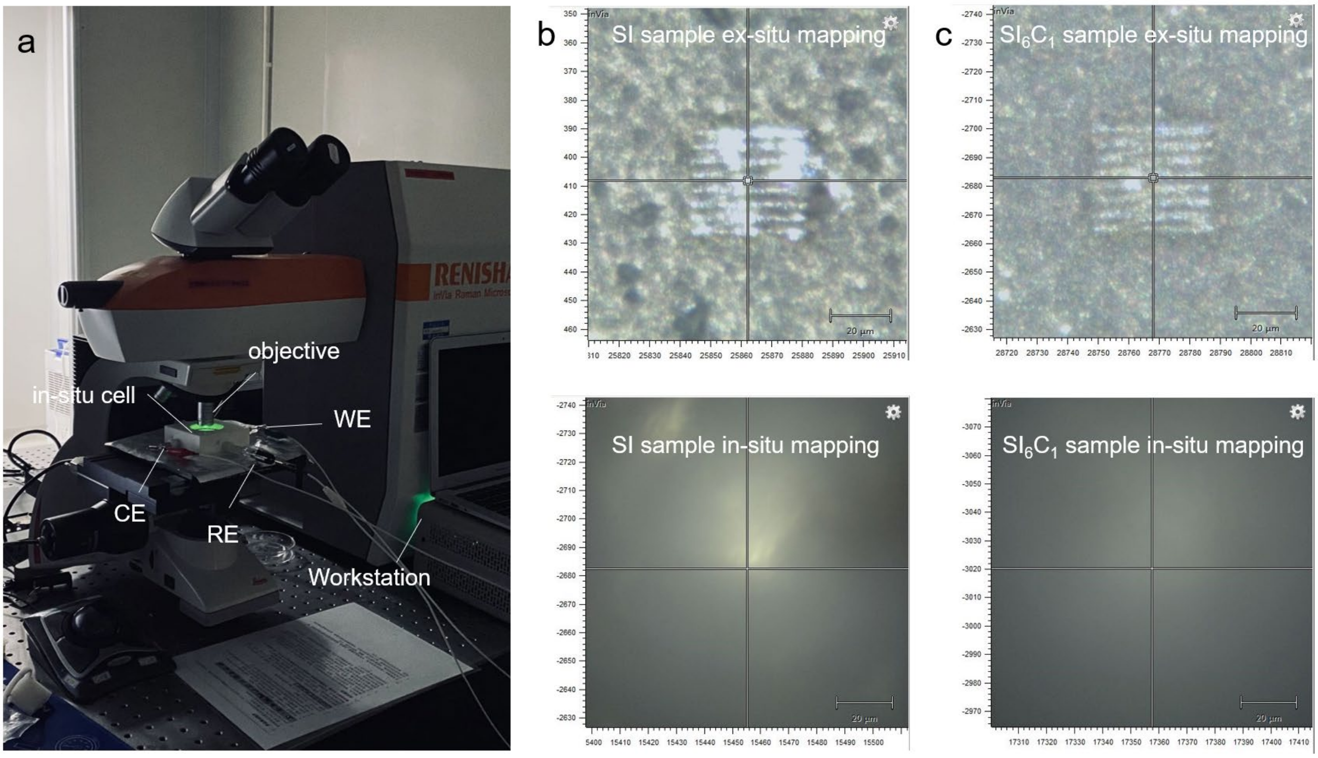

Supplementary Fig. 18 (a) In-situ Raman spectroscopic device photograph and (b-c) operating windows.

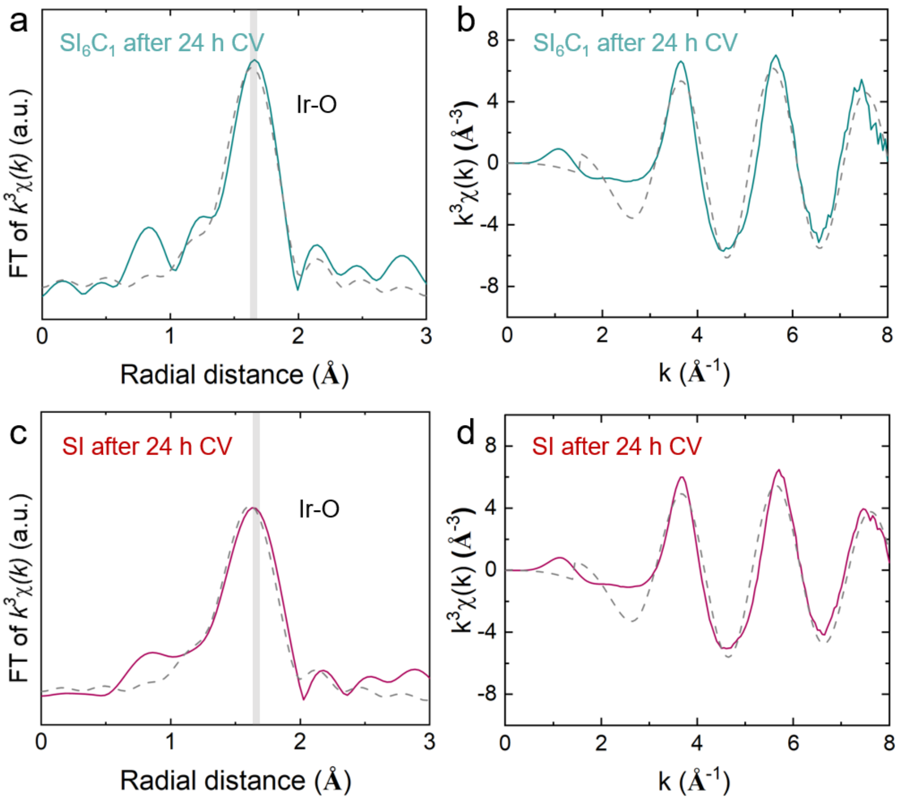

Supplementary Fig. 19 (a) χ(R) space spectra fitting curve of SI_6C_1 (after 24 h of CV); (b) $k^3\chi(k)$ space spectra fitting curve of SI_6C_1 (after 24 h of CV); (c) χ(R) space spectra fitting curve of SI (after 24 h of CV); (d) $k^3\chi(k)$ space spectra fitting curve of SI (after 24 h of CV).

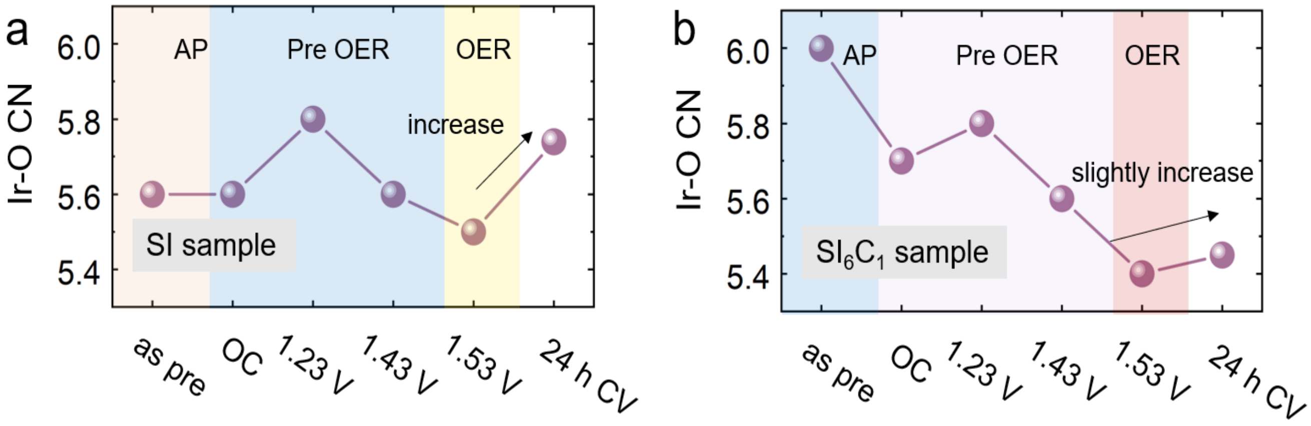

Supplementary Fig. 20 (a) Changes in Ir-O coordination of SI sample (after 24 h of CV); (b) Changes in Ir-O coordination of SI_6C_1 sample (after 24 h of CV).

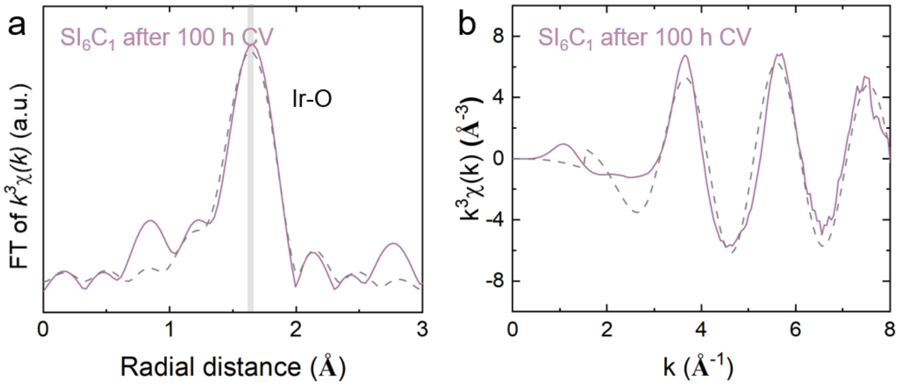

Supplementary Fig. 21 (a) χ(R) space spectra fitting curve of SI_6C_1 (after 100 h of CV); (b) $k^3\chi(k)$ space spectra fitting curve of SI_6C_1 (after 100 h of CV).

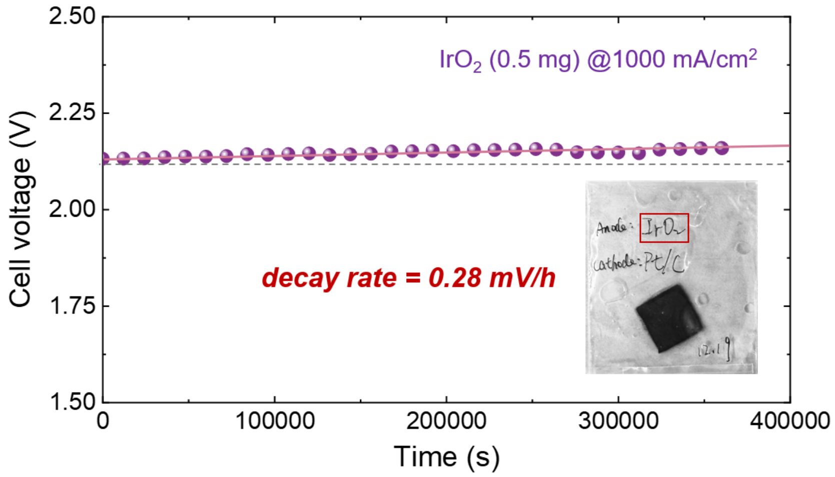

Supplementary Fig. 22 PEM water electrolysis stability of IrO_2-0.5 mg (at 1000 mA/cm^2), inset: photograph of IrO_2-0.5 mg sample for PEM water electrolysis.

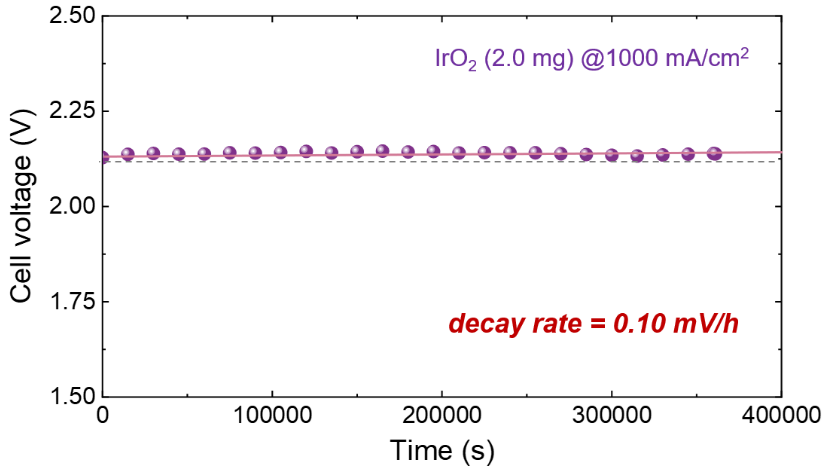

Supplementary Fig. 23 PEM water electrolysis stability of IrO_2-2.0 mg (at 1000 mA/cm^2).

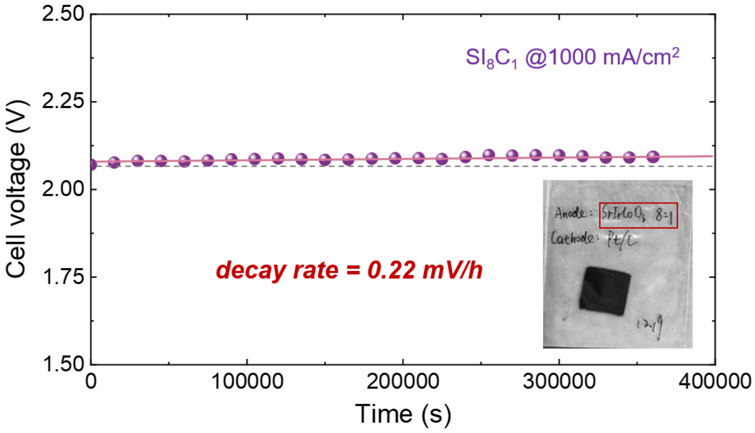

Supplementary Fig. 24 PEM water electrolysis stability of SI_8C_1 (at 1000 mA/cm^2). inset: photograph of SI_8C_1 sample for PEM water electrolysis.

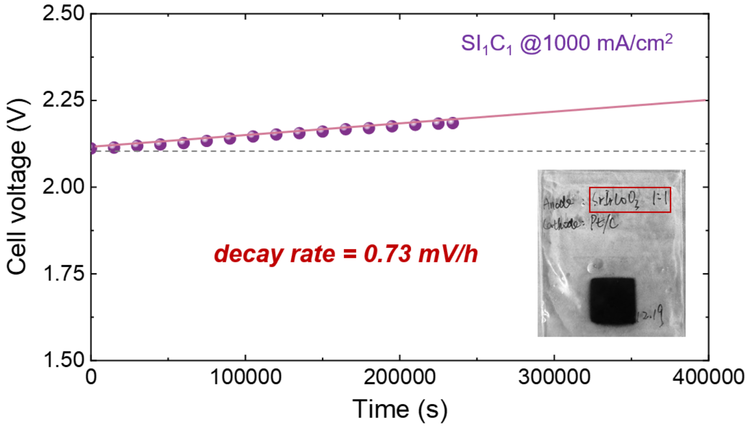

Supplementary Fig. 25 PEM water electrolysis stability of SI_1C_1 (at 1000 mA/cm^2). inset: photograph of SI_1C_1 sample for PEM water electrolysis.

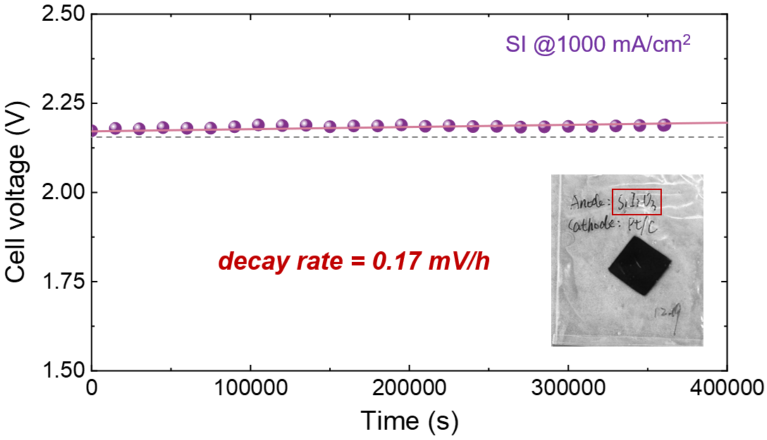

Supplementary Fig. 26 PEM water electrolysis stability of SI (at 1000 mA/cm^2). inset: photograph of SI sample for PEM water electrolysis.

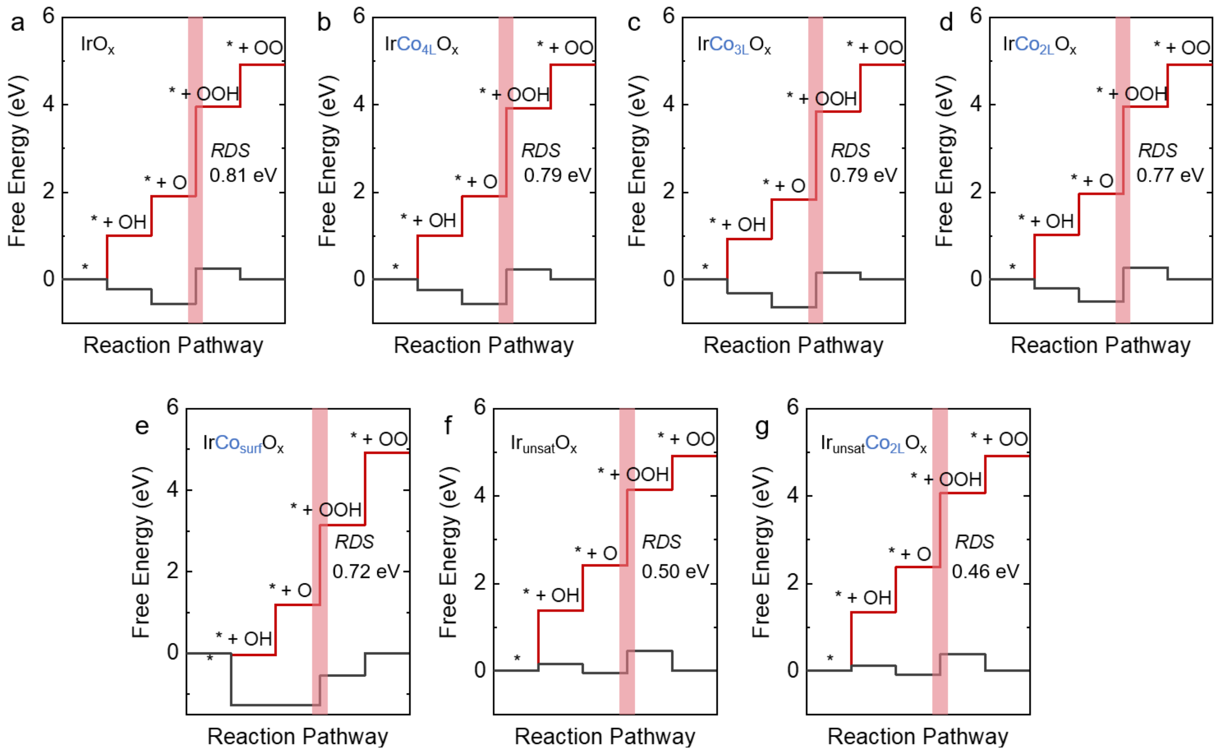

Supplementary Fig. 27 OER free energy diagrams of different models: (a) IrO_x, (b) $IrCo_{4L}O_x$, (c) $IrCo_{3L}O_x$, (d) $IrCo_{2L}O_x$, (e) $IrCo_{surf}O_x$, (f) $Ir_{unsat}Co_{2L}O_x$, and (g) $Ir_{unsat}Co_{2L}O_x$.

Supplementary Table 1 EXAFS fitting parameters of SI at the Ir L-edge

samples	path	C. N.[a]	R (Å) [b]	σ^2 (× 10^{-3} Å^2) [c]	ΔE (eV) [d]	R factor[e]
ex-situ	Ir-O	5.6	2.01	4.4	8.2	0.01
ocp	Ir-O	5.6	2.02	4.1	9.6	0.01
1.033V	Ir-O	5.8	1.98	5.4	7.4	0.02
1.233V	Ir-O	5.6	1.98	6.3	9.2	0.02
1.333V	Ir-O	5.5	1.99	9.8	9.4	0.02

[a]C. N.: coordination numbers; [b]*R*: bond distance; [c]σ^2: Debye-Waller factors; [d]ΔE_0: the inner potential correction. [e]*R* factor:

goodness of fit.

Supplementary Table 2 EXAFS fitting parameters of SI_6C_1 at the Ir L-edge

samples	path	C. N.[a]	R (Å) [b]	σ^2 (× 10^{-3} $Å^2$) [c]	ΔE (eV) [d]	R factor[e]
ex-situ	Ir-O	5.7	1.98	3.1	8.3	0.02
ocp	Ir-O	6.0	1.99	3.3	8.1	0.02
1.033V	Ir-O	5.8	1.98	5.4	8.6	0.02
1.233V	Ir-O	5.6	1.97	4.1	7.9	0.02
1.333V	Ir-O	5.4	1.97	4.6	5.9	0.02

[a]C. N.: coordination numbers; [b]*R*: bond distance; [c]σ^2: Debye-Waller factors; [d]ΔE_0: the inner potential correction. [e]*R* factor: goodness of fit.

Supplementary Table 3 EXAFS fitting parameters of samples at the Ir L-edge.

samples	path	C. N.[a]	R (Å) [b]	σ^2 (× 10^{-3} $Å^2$) [c]	ΔE (eV) [d]	R factor[e]
SI_6C_1 100 h	Ir-O	5.627	2.01	4.6	9.0	0.86%
SI_6C_1 24 h	Ir-O	5.446	2.01	5.4	9.0	0.66%
SI 24 h	Ir-O	5.743	1.99	6.6	7.8	0.94%

[a]C. N.: coordination numbers; [b]*R*: bond distance; [c]σ^2: Debye-Waller factors; [d]ΔE_0: the inner potential correction. [e]*R* factor: goodness of fit.

Supplementary Table 4 Structure parameters of IrO_x.

number	atom	x	y	z
1	O	0.20557	0.70398	0.5042
2	O	0.90536	0.0801	0.49123
3	O	0.64418	0.29791	0.46876
4	Ir	0.48304	0.32046	0.51018
5	Ir	0.85463	0.23456	0.44221
6	Ir	0.41347	0.71459	0.48651
7	Ir	0.8116	0.9152	0.45636
8	O	0.34732	0.20218	0.54002
9	O	0.50108	0.54555	0.52736
10	O	0.64032	0.77596	0.47423
11	O	0.89174	0.78112	0.40997
12	O	0.35165	0.89026	0.44029
13	O	0.09315	0.23647	0.42666
14	O	0.17831	0.04684	0.35833
15	O	0.85373	0.4044	0.3929
16	O	0.57834	0.58418	0.39449
17	Ir	0.39885	0.06479	0.33371
18	Ir	0.01983	0.23079	0.36755
19	Ir	0.38292	0.71554	0.40035
20	Ir	0.80095	0.62456	0.36824
21	O	0.19768	0.61302	0.38559
22	O	0.46227	0.84337	0.35087
23	O	0.54379	0.18906	0.36181
24	O	0.9246	0.23583	0.31219
25	O	0.3176	0.27604	0.30046
26	O	0.77342	0.75994	0.31842
27	O	0.87064	0.57366	0.25079
28	O	0.69541	0.02741	0.27111
29	O	0.35321	0.98854	0.27584
30	Ir	0.23377	0.18142	0.24899
31	Ir	0.84336	0.20728	0.25336
32	Ir	0.10769	0.60207	0.2428
33	Ir	0.7722	0.80081	0.25888
34	O	0.03698	0.05358	0.24642
35	O	0.06533	0.36273	0.2361
36	O	0.21345	0.62917	0.29227
37	O	0.77056	0.80528	0.19965
38	O	0.20047	0.74254	0.20226
39	O	0.34227	0.19133	0.19644
40	O	0.73247	0.22679	0.20067

41	O	0.00267	0.28356	0.15087
42	O	0.2972	0.50028	0.14793
43	O	0.70814	0.50028	0.14793
44	O	0.50267	0.79917	0.14676
45	Ir	0.25267	0.25000	0.13793
46	Ir	0.75267	0.25000	0.13793
47	Ir	0.25267	0.75000	0.13793
48	Ir	0.75267	0.75000	0.13793
49	O	0.00267	0.70083	0.12911
50	O	0.7972	0.99972	0.12794
51	O	0.20814	0.99972	0.12794
52	O	0.50267	0.21644	0.125
53	Sr	0.50267	0.48422	0.07899
54	Sr	0.50267	0.97412	0.07633
55	O	0.7972	0.28801	0.07226
56	O	0.20814	0.28801	0.07226
57	O	0.2972	0.71199	0.07211
58	O	0.70814	0.71199	0.07211
59	Sr	0.00267	0.02588	0.06803
60	Sr	0.00267	0.51578	0.06538

Supplementary Table 5 Structure parameters of $IrCo_{4L}O_x$.

number	atom	x	y	z
1	O	0.20696	0.70319	0.50346
2	O	0.90133	0.08303	0.49029
3	O	0.6419	0.3021	0.46783
4	Ir	0.48341	0.32259	0.51009
5	Ir	0.85209	0.23854	0.44142
6	Ir	0.41559	0.71509	0.48639
7	Ir	0.81019	0.91774	0.45504
8	O	0.35065	0.20151	0.54007
9	O	0.50213	0.54696	0.52768
10	O	0.64164	0.77737	0.47414
11	O	0.88803	0.78438	0.40822
12	O	0.35363	0.89042	0.44003
13	O	0.09174	0.23866	0.42661
14	O	0.17875	0.04682	0.35884
15	O	0.85512	0.40784	0.3917
16	O	0.57824	0.58478	0.39346
17	Ir	0.39737	0.06475	0.33351
18	Ir	0.02033	0.23231	0.3674

19	Ir	0.38324	0.71578	0.40005
20	Ir	0.79972	0.62671	0.36643
21	O	0.19709	0.61372	0.38587
22	O	0.46093	0.84288	0.35044
23	O	0.54521	0.18652	0.3615
24	O	0.9258	0.23312	0.31197
25	O	0.31777	0.27657	0.30053
26	O	0.76723	0.75453	0.31548
27	O	0.86591	0.57134	0.24822
28	O	0.69634	0.02513	0.27021
29	O	0.35566	0.9886	0.27549
30	Ir	0.2345	0.18165	0.24895
31	Ir	0.84499	0.20575	0.25317
32	Ir	0.10292	0.60244	0.24198
33	Ir	0.76704	0.79968	0.25576
34	O	0.03864	0.0528	0.24613
35	O	0.06531	0.36237	0.23608
36	O	0.20005	0.63163	0.29262
37	O	0.76583	0.80506	0.19746
38	O	0.19883	0.74369	0.20239
39	O	0.34313	0.19182	0.19639
40	O	0.73258	0.22436	0.20057
41	O	0.00267	0.28356	0.15087
42	O	0.2972	0.50028	0.14793
43	O	0.70814	0.50028	0.14793
44	O	0.50267	0.79917	0.14676
45	Ir	0.25267	0.25	0.13793
46	Ir	0.75267	0.25	0.13793
47	Ir	0.25267	0.75	0.13793
48	Co	0.75267	0.75	0.13793
49	O	0.00267	0.70083	0.12911
50	O	0.7972	0.99972	0.12794
51	O	0.20814	0.99972	0.12794
52	O	0.50267	0.21644	0.125
53	Sr	0.50267	0.48422	0.07899
54	Sr	0.50267	0.97412	0.07633
55	O	0.7972	0.28801	0.07226
56	O	0.20814	0.28801	0.07226
57	O	0.2972	0.71199	0.07211
58	O	0.70814	0.71199	0.07211
59	Sr	0.00267	0.02588	0.06803
60	Sr	0.00267	0.51578	0.06538

Supplementary Table 6 Structure parameters of $IrCo_{3L}O_x$.

number	atom	x	y	z
1	O	0.16461	0.73247	0.51523
2	O	0.86353	0.13435	0.48903
3	O	0.60511	0.35737	0.46971
4	Ir	0.47635	0.3767	0.51911
5	Ir	0.80684	0.28936	0.44045
6	Ir	0.36868	0.76094	0.49563
7	Ir	0.75536	0.96817	0.45832
8	O	0.38087	0.23088	0.55119
9	O	0.4753	0.59948	0.53668
10	O	0.5928	0.83439	0.48173
11	O	0.83153	0.82548	0.41178
12	O	0.29447	0.92705	0.4484
13	O	0.045	0.3053	0.42699
14	O	0.15266	0.08374	0.36599
15	O	0.79868	0.43659	0.38466
16	O	0.55168	0.62302	0.40735
17	Ir	0.36761	0.08904	0.33972
18	Ir	-0.01333	0.27041	0.36773
19	Ir	0.34866	0.74591	0.41185
20	Ir	0.74746	0.67081	0.36969
21	O	0.17694	0.62694	0.39598
22	O	0.43179	0.86807	0.3617
23	O	0.53227	0.19813	0.36504
24	O	0.91018	0.2441	0.31115
25	O	0.30194	0.29465	0.30374
26	O	0.75949	0.73116	0.31344
27	O	0.8767	0.58791	0.24609
28	O	0.71543	0.00825	0.27142
29	O	0.36024	1.00293	0.28208
30	Ir	0.234	0.18964	0.25132
31	Ir	0.84056	0.19648	0.25248
32	Ir	0.11124	0.60578	0.24261
33	Co	0.77935	0.79887	0.25419
34	O	0.04268	0.05397	0.24813
35	O	0.06444	0.36644	0.23618
36	O	0.21503	0.64088	0.29189
37	O	0.77531	0.8153	0.1979
38	O	0.20514	0.74476	0.20179
39	O	0.33692	0.19058	0.19725
40	O	0.7312	0.2172	0.20017

41	O	0.00267	0.28356	0.15087
42	O	0.2972	0.50028	0.14793
43	O	0.70814	0.50028	0.14793
44	O	0.50267	0.79917	0.14676
45	Ir	0.25267	0.25	0.13793
46	Ir	0.75267	0.25	0.13793
47	Ir	0.25267	0.75	0.13793
48	Ir	0.75267	0.75	0.13793
49	O	0.00267	0.70083	0.12911
50	O	0.7972	0.99972	0.12794
51	O	0.20814	0.99972	0.12794
52	O	0.50267	0.21644	0.125
53	Sr	0.50267	0.48422	0.07899
54	Sr	0.50267	0.97412	0.07633
55	O	0.7972	0.28801	0.07226
56	O	0.20814	0.28801	0.07226
57	O	0.2972	0.71199	0.07211
58	O	0.70814	0.71199	0.07211
59	Sr	0.00267	0.02588	0.06803
60	Sr	0.00267	0.51578	0.06538

Supplementary Table 7 Structure parameters of $IrCo_{2L}O_x$.

number	atom	x	y	z
1	O	0.19895	0.73348	0.50455
2	O	0.89099	0.14195	0.48826
3	O	0.64797	0.37198	0.46405
4	Ir	0.49804	0.37896	0.50899
5	Ir	0.85409	0.296	0.43752
6	Ir	0.40638	0.76362	0.48777
7	Ir	0.79622	0.97562	0.45443
8	O	0.38396	0.23807	0.53812
9	O	0.50382	0.59919	0.5295
10	O	0.63168	0.84323	0.47878
11	O	0.86185	0.84596	0.40787
12	O	0.34163	0.93256	0.44053
13	O	0.09452	0.28156	0.42459
14	O	0.18746	0.08297	0.35956
15	O	0.87064	0.45525	0.38719
16	O	0.58869	0.6306	0.39891
17	Ir	0.40737	0.09396	0.33429
18	Ir	0.02563	0.26803	0.36532

19	Ir	0.3884	0.75105	0.40336
20	Co	0.79289	0.67277	0.37264
21	O	0.21626	0.63518	0.38643
22	O	0.47478	0.87724	0.35385
23	O	0.55254	0.22498	0.35997
24	O	0.92705	0.25218	0.31058
25	O	0.32199	0.297	0.29804
26	O	0.77535	0.7361	0.31602
27	O	0.86649	0.57013	0.24676
28	O	0.70103	0.02553	0.2727
29	O	0.35781	1.0037	0.27798
30	Ir	0.23527	0.18875	0.2488
31	Ir	0.84418	0.20717	0.25299
32	Ir	0.10256	0.60448	0.24214
33	Ir	0.77139	0.79857	0.25904
34	O	0.03999	0.05697	0.24728
35	O	0.06602	0.36559	0.23391
36	O	0.19706	0.64118	0.29261
37	O	0.76993	0.8104	0.19976
38	O	0.20099	0.74348	0.20201
39	O	0.34322	0.1903	0.1962
40	O	0.73064	0.21988	0.20043
41	O	0.00267	0.28356	0.15087
42	O	0.2972	0.50028	0.14793
43	O	0.70814	0.50028	0.14793
44	O	0.50267	0.79917	0.14676
45	Ir	0.25267	0.25	0.13793
46	Ir	0.75267	0.25	0.13793
47	Ir	0.25267	0.75	0.13793
48	Ir	0.75267	0.75	0.13793
49	O	0.00267	0.70083	0.12911
50	O	0.7972	0.99972	0.12794
51	O	0.20814	0.99972	0.12794
52	O	0.50267	0.21644	0.125
53	Sr	0.50267	0.48422	0.07899
54	Sr	0.50267	0.97412	0.07633
55	O	0.7972	0.28801	0.07226
56	O	0.20814	0.28801	0.07226
57	O	0.2972	0.71199	0.07211
58	O	0.70814	0.71199	0.07211
59	Sr	0.00267	0.02588	0.06803
60	Sr	0.00267	0.51578	0.06538

Supplementary Table 8 Structure parameters of $IrCo_{surf}O_x$.

number	atom	x	y	z
1	O	0.2191	0.70667	0.50149
2	O	0.90104	0.08433	0.49122
3	O	0.63503	0.29489	0.46663
4	Ir	0.48613	0.33973	0.51042
5	Ir	0.84585	0.23202	0.4408
6	Co	0.41125	0.72974	0.4847
7	Ir	0.80734	0.91577	0.45805
8	O	0.35748	0.20502	0.53789
9	O	0.50061	0.55835	0.5241
10	O	0.63802	0.78936	0.47869
11	O	0.89076	0.77328	0.41444
12	O	0.36055	0.87853	0.43853
13	O	0.08627	0.23522	0.42726
14	O	0.18196	0.04627	0.35959
15	O	0.84893	0.40012	0.3916
16	O	0.57592	0.58316	0.39359
17	Ir	0.40058	0.06472	0.33392
18	Ir	0.0211	0.22922	0.36761
19	Ir	0.37624	0.70862	0.39762
20	Ir	0.80144	0.62589	0.36903
21	O	0.19085	0.60239	0.38624
22	O	0.46711	0.84444	0.35105
23	O	0.54656	0.1903	0.36117
24	O	0.92618	0.23189	0.31207
25	O	0.31698	0.27526	0.30074
26	O	0.77877	0.75931	0.31871
27	O	0.87215	0.57226	0.25068
28	O	0.69523	0.02597	0.27078
29	O	0.35317	0.98778	0.27628
30	Ir	0.23417	0.18062	0.24911
31	Ir	0.84354	0.20527	0.25352
32	Ir	0.10905	0.60128	0.24281
33	Ir	0.77456	0.7989	0.25923
34	O	0.03763	0.05246	0.24596
35	O	0.0667	0.36198	0.23635
36	O	0.21491	0.62992	0.29223
37	O	0.77078	0.80548	0.19992
38	O	0.20065	0.74263	0.20226
39	O	0.34223	0.19105	0.19648
40	O	0.73402	0.22693	0.20069

41	O	0.00267	0.28356	0.15087
42	O	0.2972	0.50028	0.14793
43	O	0.70814	0.50028	0.14793
44	O	0.50267	0.79917	0.14676
45	Ir	0.25267	0.25	0.13793
46	Ir	0.75267	0.25	0.13793
47	Ir	0.25267	0.75	0.13793
48	Ir	0.75267	0.75	0.13793
49	O	0.00267	0.70083	0.12911
50	O	0.7972	0.99972	0.12794
51	O	0.20814	0.99972	0.12794
52	O	0.50267	0.21644	0.125
53	Sr	0.50267	0.48422	0.07899
54	Sr	0.50267	0.97412	0.07633
55	O	0.7972	0.28801	0.07226
56	O	0.20814	0.28801	0.07226
57	O	0.2972	0.71199	0.07211
58	O	0.70814	0.71199	0.07211
59	Sr	0.00267	0.02588	0.06803
60	Sr	0.00267	0.51578	0.06538

Supplementary Table 9 Structure parameters of $Ir_{unsat}O_x$.

number	atom	x	y	z
1	O	0.76095	0.62429	0.50783
2	O	0.29565	0.65149	0.55634
3	Ir	0.15098	0.25961	0.45508
4	Ir	0.86693	0.19294	0.49526
5	Ir	0.27203	0.63095	0.50071
6	Ir	0.65585	0.67707	0.46107
7	O	0.08707	0.16031	0.51258
8	O	0.72966	0.31192	0.5259
9	O	0.13817	0.49837	0.46755
10	O	0.42978	0.75573	0.46761
11	O	0.74384	0.67095	0.40666
12	O	0.27977	0.06859	0.43772
13	O	0.76156	0.05542	0.455
14	O	-0.08974	0.01272	0.35973
15	O	0.23811	0.31123	0.395
16	O	0.76307	0.32342	0.40169
17	O	0.48351	0.49826	0.35888
18	Ir	0.39525	0.10653	0.38469

19	Ir	0.74172	0.09397	0.38893
20	Ir	0.24351	0.50879	0.3566
21	Ir	0.74358	0.50153	0.35733
22	O	1.0053	0.50791	0.35693
23	O	0.55468	-0.06676	0.39072
24	O	0.38522	0.13678	0.32822
25	O	0.25608	0.68472	0.31582
26	O	0.73742	0.68443	0.31431
27	O	0.99432	0.79524	0.26527
28	O	0.08263	0.18052	0.27886
29	O	0.4469	0.40665	0.25226
30	Ir	0.2146	0.30564	0.24853
31	Ir	0.65099	0.29459	0.25442
32	Ir	0.25988	0.74341	0.25728
33	Ir	0.76095	0.75883	0.25871
34	O	-0.27222	0.32521	0.30931
35	O	0.13674	0.51716	0.24053
36	O	0.49993	0.77073	0.25689
37	O	0.77029	0.78636	0.20017
38	O	0.23404	0.78885	0.19904
39	O	0.29828	0.19583	0.19918
40	O	0.70054	0.23276	0.19997
41	O	0.00267	0.28356	0.15087
42	O	0.2972	0.50028	0.14793
43	O	0.70814	0.50028	0.14793
44	O	0.50267	0.79917	0.14676
45	Ir	0.25267	0.25	0.13793
46	Ir	0.75267	0.25	0.13793
47	Ir	0.25267	0.75	0.13793
48	Ir	0.75267	0.75	0.13793
49	O	0.00267	0.70083	0.12911
50	O	0.7972	0.99972	0.12794
51	O	0.20814	0.99972	0.12794
52	O	0.50267	0.21644	0.125
53	Sr	0.50267	0.48422	0.07899
54	Sr	0.50267	0.97412	0.07633
55	O	0.7972	0.28801	0.07226
56	O	0.20814	0.28801	0.07226
57	O	0.2972	0.71199	0.07211
58	O	0.70814	0.71199	0.07211
59	Sr	0.00267	0.02588	0.06803
60	Sr	0.00267	0.51578	0.06538

Supplementary Table 10 Structure parameters of $Ir_{unsat}Co_{2L}O_x$ sample.

number	atom	x	y	z
1	O	0.29249	0.50063	0.42038
2	O	0.82446	0.13579	0.49826
3	O	0.64228	0.40184	0.46599
4	Ir	0.58029	0.46498	0.52287
5	Ir	0.82491	0.26266	0.44233
6	Ir	0.68981	0.99281	0.465
7	O	0.48306	0.31202	0.55216
8	O	0.63196	0.66914	0.53321
9	O	0.47475	0.99999	0.47171
10	O	0.78868	0.82211	0.44098
11	O	0.056	0.18636	0.4296
12	O	0.13601	0.01615	0.35745
13	O	0.87571	0.4141	0.39434
14	O	0.60842	0.54823	0.36781
15	Ir	0.35587	0.03583	0.33466
16	Ir	0.99012	0.20563	0.37016
17	Ir	0.39927	0.6153	0.38242
18	Co	0.8273	0.60348	0.36032
19	O	0.01499	0.68003	0.36539
20	O	0.39433	0.81194	0.35481
21	O	0.51989	0.13211	0.36424
22	O	0.90468	0.22378	0.31377
23	O	0.30174	0.25608	0.30563
24	O	0.76932	0.75191	0.3161
25	O	0.86984	0.5713	0.24864
26	O	0.68285	0.02242	0.26792
27	O	0.349	0.97345	0.27504
28	Ir	0.23024	0.17647	0.25013
29	Ir	0.83645	0.20259	0.25426
30	Ir	0.1066	0.59889	0.24197
31	Ir	0.76621	0.79791	0.25865
32	O	0.03217	0.05259	0.24644
33	O	0.06491	0.35961	0.23887
34	O	0.21481	0.62818	0.29091
35	O	0.76719	0.80552	0.19968
36	O	0.19786	0.74206	0.20187
37	O	0.33676	0.18955	0.19691
38	O	0.73352	0.22433	0.20039
39	O	0.00267	0.28356	0.15087
40	O	0.2972	0.50028	0.14793

41	O	0.70814	0.50028	0.14793
42	O	0.50267	0.79917	0.14676
43	Ir	0.25267	0.25000	0.13793
44	Ir	0.75267	0.25000	0.13793
45	Ir	0.25267	0.75000	0.13793
46	Ir	0.75267	0.75000	0.13793
47	O	0.00267	0.70083	0.12911
48	O	0.7972	0.99972	0.12794
49	O	0.20814	0.99972	0.12794
50	O	0.50267	0.21644	0.125
51	Sr	0.50267	0.48422	0.07899
52	Sr	0.50267	0.97412	0.07633
53	O	0.7972	0.28801	0.07226
54	O	0.20814	0.28801	0.07226
55	O	0.2972	0.71199	0.07211
56	O	0.70814	0.71199	0.07211
57	Sr	0.00267	0.02588	0.06803
